# Folate transporter dynamics and therapy with classic and tumor-targeted antifolates

**DOI:** 10.1038/s41598-021-85818-x

**Published:** 2021-03-18

**Authors:** Carrie O’Connor, Adrianne Wallace-Povirk, Changwen Ning, Josephine Frühauf, Nian Tong, Aleem Gangjee, Larry H. Matherly, Zhanjun Hou

**Affiliations:** 1 Departments of Oncology, Wayne State University School of Medicine, 421 E. Canfield, Detroit, MI 48201 USA; 2 Department of Pharmacology, Wayne State University School of Medicine, Detroit, MI USA; 3 Molecular Therapeutics Program, Barbara Ann Karmanos Cancer Institute, Detroit, MI USA; 4 Division of Medicinal Chemistry, Duquesne University, Pittsburgh, PA USA

**Keywords:** Cancer, Drug discovery

## Abstract

There are three major folate uptake systems in human tissues and tumors, including the reduced folate carrier (RFC), folate receptors (FRs) and proton-coupled folate transporter (PCFT). We studied the functional interrelationships among these systems for the novel tumor-targeted antifolates AGF94 (transported by PCFT and FRs but not RFC) and AGF102 (selective for FRs) versus the classic antifolates pemetrexed, methotrexate and PT523 (variously transported by FRs, PCFT and RFC). We engineered HeLa cell models to express FRα or RFC under control of a tetracycline-inducible promoter with or without constitutive PCFT. We showed that cellular accumulations of extracellular folates were determined by the type and levels of the major folate transporters, with PCFT and RFC prevailing over FRα, depending on expression levels and pH. Based on patterns of cell proliferation in the presence of the inhibitors, we established transport redundancy for RFC and PCFT in pemetrexed uptake, and for PCFT and FRα in AGF94 uptake; uptake by PCFT predominated for pemetrexed and FRα for AGF94. For methotrexate and PT523, uptake by RFC predominated even in the presence of PCFT or FRα. For both classic (methotrexate, PT523) and FRα-targeted (AGF102) antifolates, anti-proliferative activities were antagonized by PCFT, likely due to its robust activity in mediating folate accumulation. Collectively, our findings describe a previously unrecognized interplay among the major folate transport systems that depends on transporter levels and extracellular pH, and that determines their contributions to the uptake and anti-tumor efficacies of targeted and untargeted antifolates.

## Introduction

Folates are hydrophilic molecules that are essential for life and folate deficiency contributes to cardiovascular disease, fetal abnormalities, neurologic disorders, and cancer^1–5^. Since mammals cannot synthesize folates de novo, their cellular uptake from the extracellular milieu is essential. Uptake of folates into mammalian cells is principally mediated by facilitative transporters, the reduced folate carrier (RFC) and proton-coupled folate transporter (PCFT), and by folate receptors (FRs)^6–8^.

These uptake systems are genetically distinct and functionally diverse, and each plays a unique role in mediating folate uptake across epithelia and into systemic tissues^6–8^. RFC is ubiquitously expressed and is the major tissue folate transporter^7^. PCFT was identified as a high affinity folate-proton symporter in the upper gastrointestinal (GI) tract^9^ and in tumors^10–14^, whereby it transports folates and related molecules under acidic conditions^15–17^. FRs are glycosylphosphatidylinositol-anchored proteins that function by endocytosis^6^. The major folate receptor (FR) α isoform is expressed in a number of normal tissues such as kidney, lung, choroid plexus and placenta^18^, and in ovarian, lung, and breast cancers^12,19–21^.

Following internalization, folates participate in one-carbon (C1) transfers leading to synthesis of thymidylate, purines, serine and methionine, and in biological methylation reactions from S-adenosyl methionine^22^. C1 metabolism encompasses cytosolic and mitochondrial pathways connected by interchanges between serine, glycine and formate^22–24^. Antifolate drugs target these pathways for treating cancers and other diseases, typified by methotrexate (MTX) and pemetrexed (PMX)^25^. Key cytosolic C1 enzymes such as dihydrofolate reductase, thymidylate synthase, and the purine biosynthetic enzymes β-glycinamide ribonucleotide formyltransferase (GARFTase) and 5-aminoimidazole-4-carboxamide ribonucleotide formyltransferase (AICARFTase) are important targets for cancer therapy with these inhibitors^25,26^.

**Figure 1:**
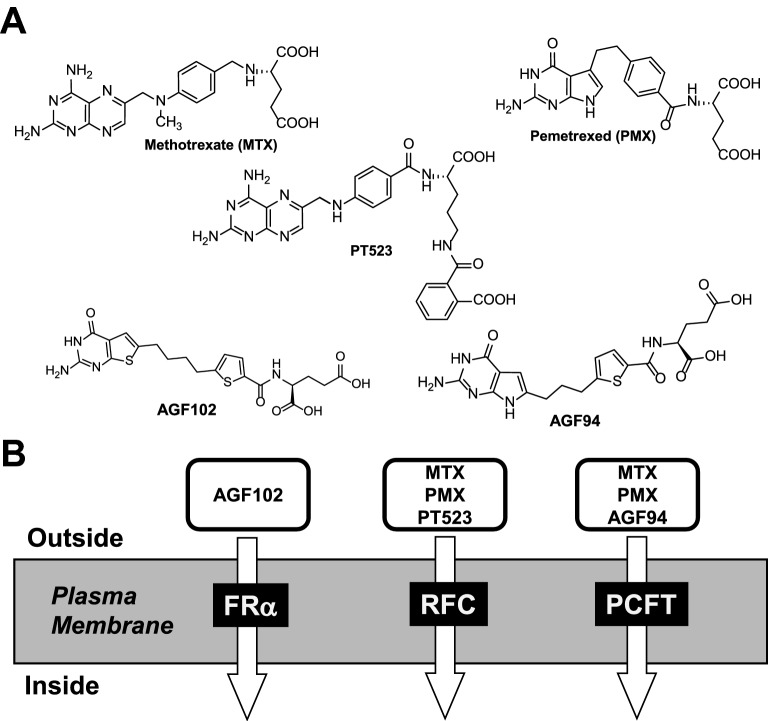
(**A**) Structures of MTX, PMX, PT523, AGF94, and AGF102. (**B**) A cartoon depicting three folate transport systems, with their transport substrates noted.

Classic antifolates (e.g., MTX, PMX; Fig. 1) are substrates for RFC, with varying transport via PCFT and FRs^7,27^. Facilitated uptake is essential to ensuring sufficient intracellular drug to inhibit enzyme targets and drive synthesis of antifolate polyglutamate conjugates, and to effect cytotoxic and therapeutic responses^25,27^. However, as RFC substrates, these compounds show limited selectivity toward tumors over proliferative tissues (e.g., bone marrow) as tumors and normal tissues all express RFC^7,27^.

On this basis, we reasoned if we could design inhibitors of C1 metabolism that target tumors via FRs and PCFT, these would exhibit greater potencies and selectivity than classic drugs such as PMX or MTX^15,16,28,29^. For FRα, additional selectivity would result from its non-polarized localization in tumors where FRs are accessible to the circulation, while in normal (polarized) tissues FRα is localized to luminal membranes^6^. As PCFT is expressed in tumors (above) and expression is limited in normal tissues that do not experience acidic microenvironments favoring PCFT transport^9,16,17,30,31^, cytotoxic PCFT transport substrates should be selectively active toward tumors^15,16,27^.

We discovered 6-substituted pyrrolo[2,3-*d*]pyrimidine inhibitors typified by AGF94 (Fig. 1) with selectivity for FRs and PCFT over RFC^15,16,28^. Toward malignant pleural mesothelioma, lung adenocarcinoma and ovarian cancer models, AGF94 inhibited cell proliferation, all or in part via its uptake by PCFT and/or FRα^12,14,32^. Upon internalization, AGF94 is metabolized to polyglutamates that inhibit GARFTase, the first folate-dependent step in de novo purine biosynthesis^28,32,33^. In vivo antitumor efficacies were demonstrated for AGF94 with H2452 mesothelioma^32^, H460 lung adenocarcinoma^14^ and IGROV1 epithelial ovarian cancer^12^ xenografts. Replacement of the pyrrole with a thiophene results in thieno[2,3-*d*]pyrimidine inhibitors (e.g., AGF102) (Fig. 1) with absolute FR selectivity over either PCFT or RFC, accompanied by dual inhibition of purine biosynthesis at both GARFTase and AICARFTase^34^.

While PCFT is functionally distinct from FRα and RFC, an important consideration in implementing novel FR- and PCFT-targeted therapeutics for cancer involves the complex interplay among these uptake systems that manifests as cooperativity, redundancy or even antagonism, depending on transporter specificities, levels of expression, and cellular context. For instance, the impact of loss of RFC on MTX efficacy can be profound in spite of the presence of other transporters, but is less so for PMX which is a much better PCFT substrate^16,27^. The impact of loss of FRα on AGF94 anti-tumor sensitivity depends on the presence of PCFT and vice versa^12^. Further, the presence of RFC can influence potencies of PCFT-targeted agents such as AGF94 that are not themselves RFC substrates, by mediating accumulation of cellular tetrahydrofolate cofactors that compete for polyglutamylation and/or binding to intracellular targets^33^. A similar effect could manifest for PCFT and impact efficacy of RFC or FR cytotoxic substrates. Of course, all these effects would be influenced by extracellular pH such that acid pH (approximating the tumor microenvironment) favors PCFT uptake over other processes^16,27^.

As these interrelationships are not predictable and are often not intuitive, we engineered novel tumor cell line models from a PCFT-, FR-, and RFC-null HeLa cell background to express FRα or RFC under control of a tetracycline (Tet)-inducible promoter to systematically assess these interactions. We constitutively expressed PCFT in this context, to explore folate transport cooperativity, redundancy and/or antagonism between RFC and PCFT, and between FRα and PCFT, as reflected in in vitro efficacies of FR- and/or PCFT-targeted (AGF94, AGF102) and untargeted (MTX, PMX) antifolates.

## Results

FRα expression in different cancers spans a wide range^12,18–21^. RFC is ubiquitously expressed, although levels can vary, and in MTX resistant tumors, loss of RFC occurs and contributes to the drug resistant phenotype^7^. PCFT is widely expressed among solid tumors and less so in normal tissues^15–17,30^. Our goal was to *systematically* study the impact of constitutive expression of PCFT in relation to levels of FRα or RFC on folate homeostasis and on sensitivities to classic and to PCFT and FR-targeted antifolates. Accordingly, we expressed FRα and RFC in engineered HeLa-derived models over a wide range spanning levels commonly found in tumor cells.

### FRα and PCFT

We engineered a cell line model based on a PCFT-, FR-, and RFC-null HeLa (R1-11) background to express FRα (designated R1-11/Tet-on-FRα) under control of a Tet-inducible promoter. We constitutively expressed PCFT [HSV-thymidine kinase (TK) promoter] in these cells (R1-11/Tet-on-FRα/PCFT). Both FRα and PCFT proteins were hemagglutinin (HA)-tagged for detection on Western blots with HA-specific antibody.

**Figure 2:**
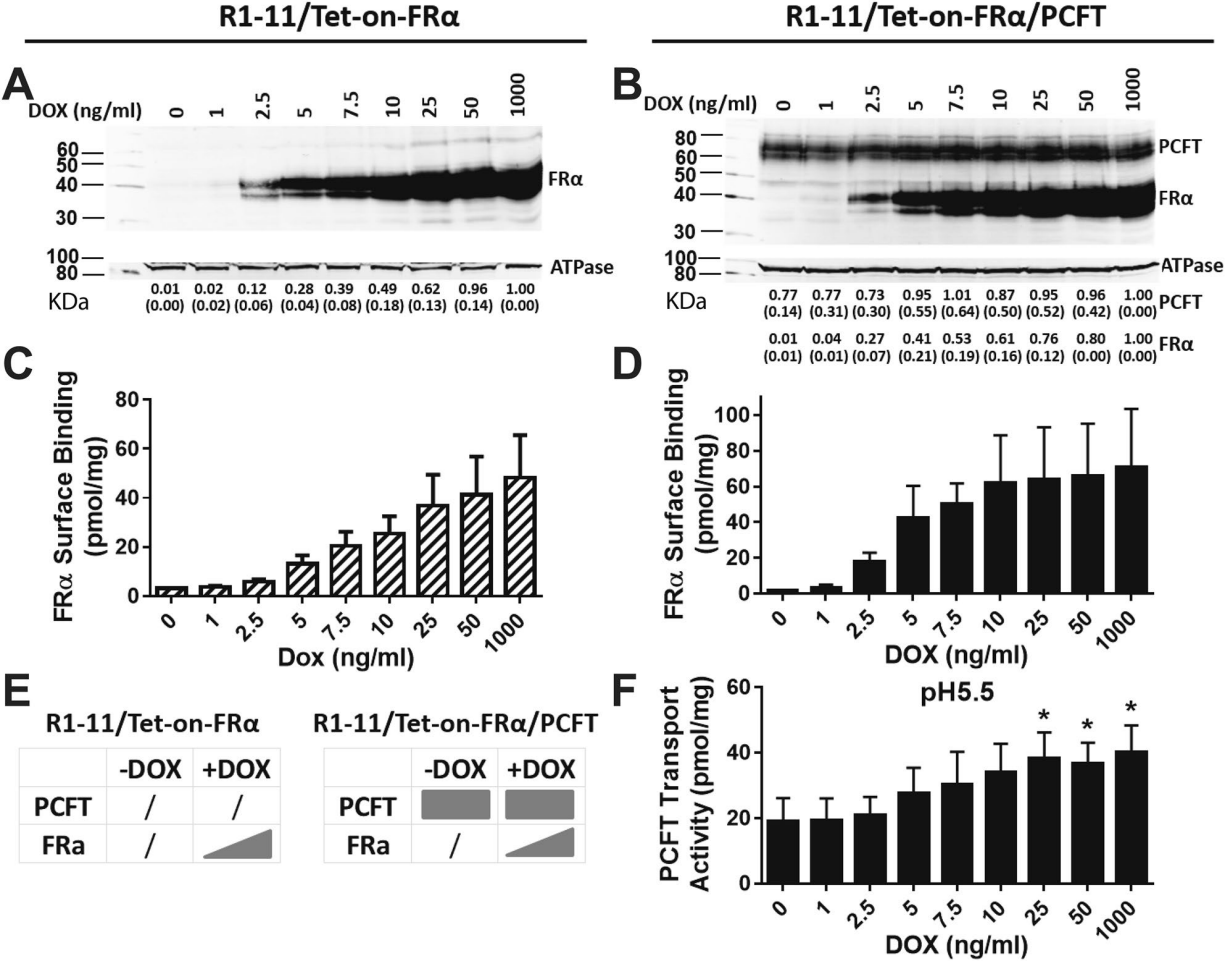
*Characterization of R1-11/Tet-on-FRα single and R1-11/Tet-on-FRα/PCFT double transfectant models.* R1-11/Tet-on-FRα or R1-11/Tet-on-FRα/PCFT cells were plated in 60-mm dishes in complete folate-free (FF) RPMI 1640 medium containing 10% fetal bovine serum (FBS) for transport/binding and protein expression assays. Twenty-four hours later, a range of DOX (0, 1, 2.5, 5, 7.5, 10, 25, 50, and 1000 ng/ml) was added. After 48 h, FRα and/or PCFT protein levels for the R1-11/Tet-on-FRα single (**A**) or R1-11/Tet-on-FRα/PCFT double (**B**) models were measured in crude plasma membranes by SDS-PAGE and Western blotting with a HA monoclonal antibody (upper panels) followed by stripping and re-probing with Na^+^/K^+^ ATPase monoclonal antibody (lower panels) as a loading control. Blots were cropped as needed. The full blots are included in the Supplement (Figs. S2–S5). The molecular mass markers for SDS-PAGE are noted. Densitometry was performed using Odyssey software, and FRα or PCFT protein levels were normalized to those for Na^+^/K^+^ ATPase and expressed relative to the level at the maximum concentration of DOX. Densitometry results are summarized below the individual lanes and are presented as mean values plus/minus standard deviations (SDs; in parenthesis) from at least 3 experiments. FRα levels (**C**,**D**) were also determined with [^3^H]FA at 0 °C for 15 min; PCFT uptake (**F**) was measured with [^3^H]MTX at pH 5.5 at 37 °C for 2 min. Results are presented as mean values plus/minus SDs from at least 3 experiments. The statistical significance of PCFT transport activities between samples with and without DOX was analyzed by an unpaired t test. An asterisk indicates a statistically significant difference between the mean transport values (*p* < 0.05). A schematic is shown depicting the patterns of expression of FRα in R1-11/Tet-on-FRα cells, and of FRα and/or PCFT in R1-11/Tet-on-FRα/PCFT cells, in the presence or absence of DOX (**E**).

R1-11/Tet-on-FRα and R1-11/Tet-on-FRα/PCFT cells were treated with a range of doxycycline (DOX) up to 1000 ng/ml for 48 h (Fig. 2E), and FRα and PCFT proteins were analyzed on Westerns (Fig. 2A,B). For both the R1-11/Tet-on-FRα and R1-11/Tet-on-FRα/PCFT cells, FRα showed a nearly identical increase with DOX that was maximal at 1000 ng/ml. In R1-11/Tet-on-FRα/PCFT double-transfected cells, PCFT protein levels were relatively steady, although a subtle (~ 20%) increase in PCFT was detected up to 5 ng/ml DOX that was unchanged up to 1000 ng/ml DOX.

As a functional read-out of FRα levels in R1-11/Tet-on-FRα and R1-11/Tet-on-FRα/PCFT cells, we measured binding of [^3^H]folate acid (FA) to surface FRα at 0–4 °C^12^ (Fig. 2C,D). Levels of [^3^H]FA surface binding paralleled changes in FRα on Westerns and were not statistically different between the two cell lines. We assayed PCFT transport over 2 min with [^3^H]MTX at pH 5.5, the PCFT pH optimum^8,35^. Substantial transport was detected. Interestingly, transport activity for PCFT increased ~ 2-fold from 0 to 25 ng/ml DOX (Fig. 2F). This exceeds the modest change in PCFT protein detected with increasing DOX (Fig. 2B).

### RFC and PCFT

To extend studies to PCFT and RFC, we engineered R1-11 HeLa cells to express RFC under control of a Tet-inducible promoter (R1-11/Tet-on-RFC). We constitutively expressed PCFT in the R1-11/Tet-on-RFC cells to generate the R1-11/Tet-on-RFC/PCFT subline. Both RFC and PCFT proteins were HA-tagged for detection on Westerns. As both RFC and PCFT are glycosylated^36^, for simultaneous detection on Westerns, samples were deglycosylated with N-glycosidase F prior to fractionation and analysis.

**Figure 3:**
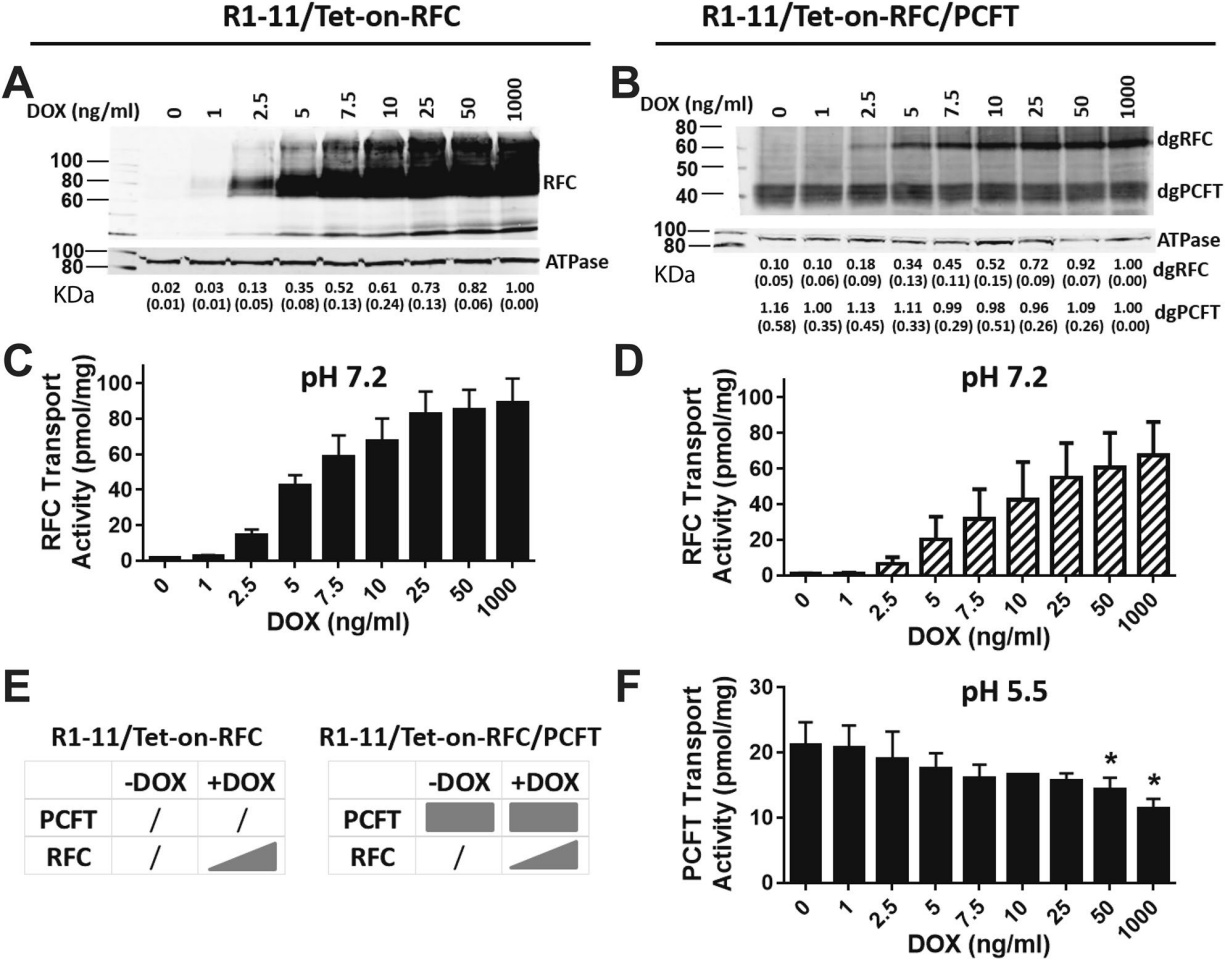
*Characterization of R1-11/Tet-on-RFC single and R1-11/Tet-on-RFC/PCFT double transfectant models.* R1-11/Tet-on-RFC or R1-11/Tet-on-RFC/PCFT cells were plated in 60-mm dishes in complete FF RPMI 1640 medium containing 10% FBS for transport and protein expression assays. Twenty-four hours later, a range of DOX (0, 1, 2.5, 5, 7.5, 10, 25, 50, and 1000 ng/ml) was added. After 48 h, RFC and PCFT protein levels for the R1-11/Tet-on-RFC (**A**) and R1-11/Tet-on-RFC/PCFT models (after deglycosylation; “dgRFC” and “dgPCFT” designate the deglycosylated forms of these proteins) (**B**) were measured in crude plasma membranes by SDS-PAGE and Western blotting with HA monoclonal antibody (upper panels), followed by stripping and re-probing with Na^+^/K^+^ ATPase monoclonal antibody (lower panels) as a loading control. Blots were cropped as needed. The full blots are included in the Supplement (Figs. S6 to S9). The molecular mass markers for SDS-PAGE are noted. Densitometry was performed using the Odyssey software, and RFC or PCFT protein levels were normalized to Na^+^/K^+^ ATPase and expressed relative to the level at the maximum concentration of DOX. Densitometry results are noted below the individual lanes and are presented as mean values plus/minus SDs from at least 3 experiments. RFC (**C**,**D**) and PCFT (**F**) transport activities were measured with [^3^H]MTX at 37 °C for 2 min, at pH 7.2 and pH 5.5, respectively. Results are presented as mean values plus/minus SDs from at least 3 experiments. Statistical significance of PCFT transport activities between samples with and without DOX was analyzed by the unpaired t test. An asterisk indicates a statistically significant difference between the mean values of PCFT transport (*p* < 0.05). A schematic is shown depicting the expression of RFC in R1-11/Tet-on-RFC cells, and RFC and/or PCFT in R1-11/Tet-on-RFC/PCFT cells, in the presence or absence of DOX (**E**).

Treatment of R1-11/Tet-on-RFC and R1-11/Tet-on-RFC/PCFT cells with 0–1000 ng/ml DOX for 48 h (Fig. 3E) resulted in a similar pattern of induction of RFC protein (Fig. 3A,B). [^3^H]MTX transport (2 min, 37 °C) at pH 7.2 (measures RFC without PCFT) (Fig. 3C,D) between the cell lines was likewise similar and paralleled levels of RFC protein. To assay PCFT function in the R1-11/Tet-on-RFC/PCFT double-transfected cells, [^3^H]MTX uptake was assayed over 2 min at pH 5.5 (Fig. 3F), conditions under which RFC is largely inactive^7^. Although PCFT protein on Westerns was constant (Fig. 3B), PCFT transport decreased ~ 50% with increasing RFC up to 1000 ng/ml DOX (Fig. 3F).

### pH dependent transport for dual RFC/PCFT R1-11/Tet-on-RFC/PCFT cells

**Figure 4:**
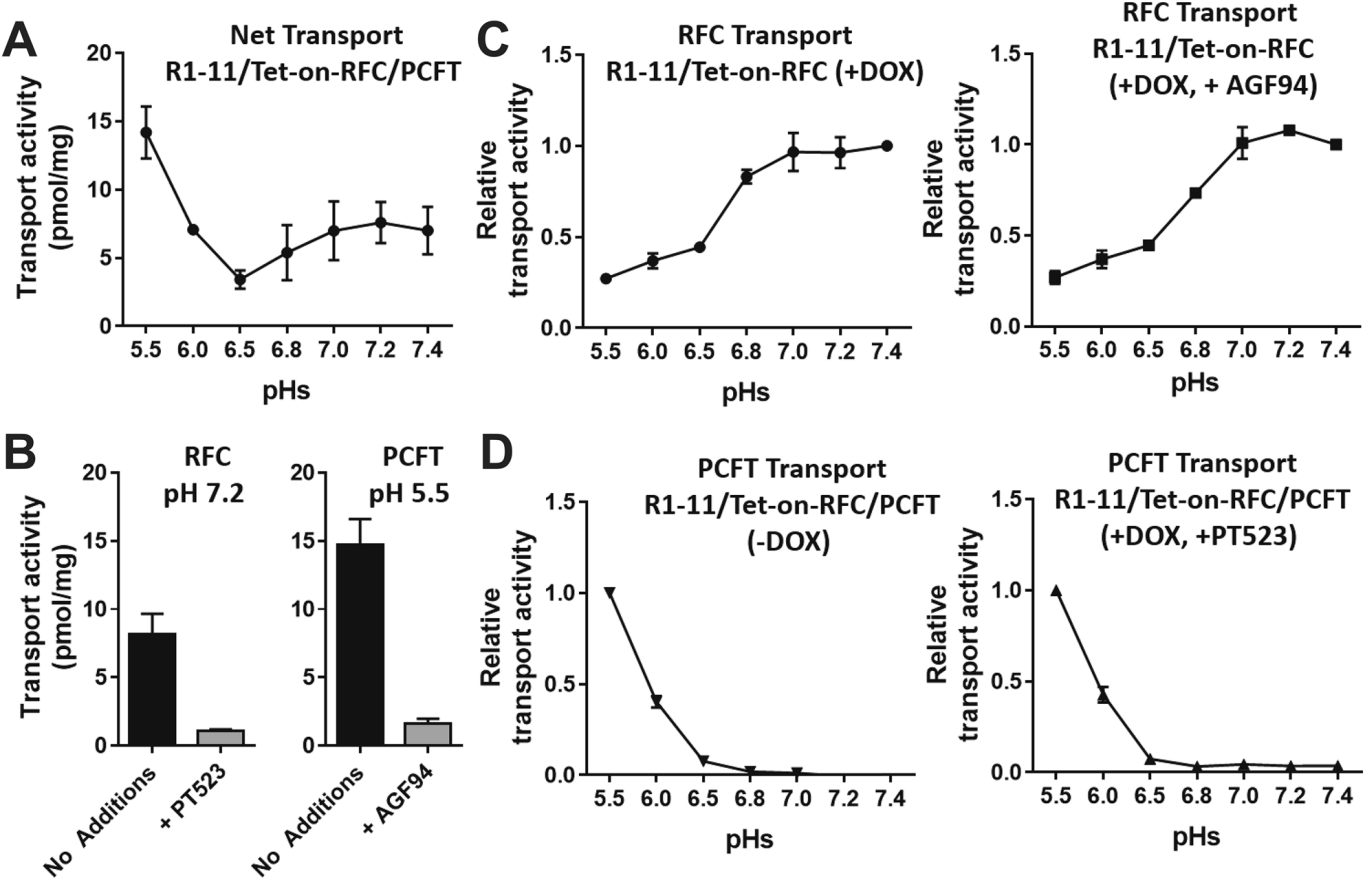
*Transport pH profiling of R1-11/Tet-on-RFC single and R1-11/Tet-on-RFC/PCFT double models.* R1-11/Tet-on-RFC or R1-11/Tet-on-RFC/PCFT cells were plated in 60-mm dishes in complete FF RPMI 1640 medium containing 10% FBS. Twenty-four hours later, DOX was added at 10 ng/ml. After 48 h, transport was measured over 2 min at 37 °C with [^3^H]MTX (0.5 µM) in MBS (20 mM MES, 140 mM NaCl, 5 mM KCl, 2 mM MgCl_2_, and 5 mM glucose, for pH 5.5, 6.0 and 6.5) and HBS (20 mM Hepes, 140 mM NaCl, 5 mM KCl, 2 mM MgCl_2_, and 5 mM glucose, for pH 6.8, 7.0, 7.2 and 7.4). The dishes were washed (3×) with ice-cold PBS. The cells were solubilized in 0.5 N NaOH, and the radioactive contents and protein concentrations of the alkaline cell homogenates were determined. Intracellular radioactivity is calculated in units of pmol [^3^H]MTX per mg of cell protein and results are presented as mean values plus/minus SDs (Panel A, B) or relative values (relative to the maximum activity for each transporter) as mean plus/minus SDs (Panel C, D) from at least 3 experiments. (**A**) Net transport activities of R1-11/Tet-on-RFC/PCFT cells (in the presence of DOX) are shown over a range of pHs. (**B**) The results depict transport activities of R1-11/Tet-on-RFC/PCFT cells at pH 7.2 (optimum for RFC transport) in the absence or presence of 10 μM PT523 (left panel), and at pH 5.5 (optimum for PCFT transport) in the absence or presence of 10 μM AGF94 (right panel). (**C**) RFC transport activities of R1-11/Tet-on-RFC cells (with DOX; left panel) and of R1-11/Tet-on-RFC/PCFT cells (with DOX and in the presence of PCFT specific inhibitor AGF94 of 10 μM; right panel) are shown over a range of pHs. (**D**) PCFT transport activities of R1-11/Tet-on-RFC/PCFT cells (without DOX; left panel) and of R1-11/Tet-on-RFC/PCFT cells (with DOX and in the presence of RFC specific inhibitor PT523 at 10 μM; right panel) are shown over a range of pHs.

For the R1-11/Tet-on-RFC/PCFT dual-transfected cells, we profiled RFC and PCFT transport activity from pH 5.5–7.4 with [^3^H]MTX in the presence of DOX (10 ng/ml) (Fig. 4). Net transport showed an unusual bimodal pH profile with distinct pH maxima at pH 5.5 and pH 7.2, and a nadir at pH 6.5 (Fig. 4A).

To identify the individual (RFC versus PCFT) transport fluxes, assays were performed in the presence of 10 μM of the RFC [N^α^-(4-amino-4-deoxypteroyl)-N^δ^-hemiphthaloyl-L-ornithine (PT523)]^37^ or PCFT (AGF94)^28^ inhibitors. PT523 selectively inhibits RFC transport^38^ and AGF94 selectively inhibits PCFT transport^13,32,33^. Results for R1-11/Tet-on-RFC/PCFT cells are shown in Fig. 4B.

Results are presented for RFC transport in R1-11/Tet-on-RFC cells (no PCFT) (Fig. 4C; left panel) which paralleled those for R1-11/Tet-on-RFC/PCFT cells treated with AGF94 (Fig. 4C; right panel). While transport showed a distinct optimum at pH 7.2–7.4, discernible RFC transport activity was detected at the lowest pH values for both the single and double transfected cells. In uninduced R1-11/Tet-on-RFC/PCFT cells (PCFT only), pH-dependent PCFT transport of [^3^H]MTX was identical to that for DOX-induced R1-11/Tet-on-RFC/PCFT cells treated with PT523 (Fig. 4D).

### Role of PCFT in cellular accumulation of extracellular folates compared to FRα and RFC

RFC is the major tissue folate transporter^7,17^, reflecting its ubiquitous tissue expression and neutral pH optimum (above). Whereas PCFT is important for folate internalization at the acid pH of the upper GI^9,17,30^, its contribution to net folate accumulation versus FRα and RFC over a wide range of pH values [including those associated with the tumor microenvironment (e.g., pH 6.5–6.8)^39,40^ or accompanying cell proliferation in culture (pH ~ 6.7–6.8)^41^] is not established.

**Figure 5:**
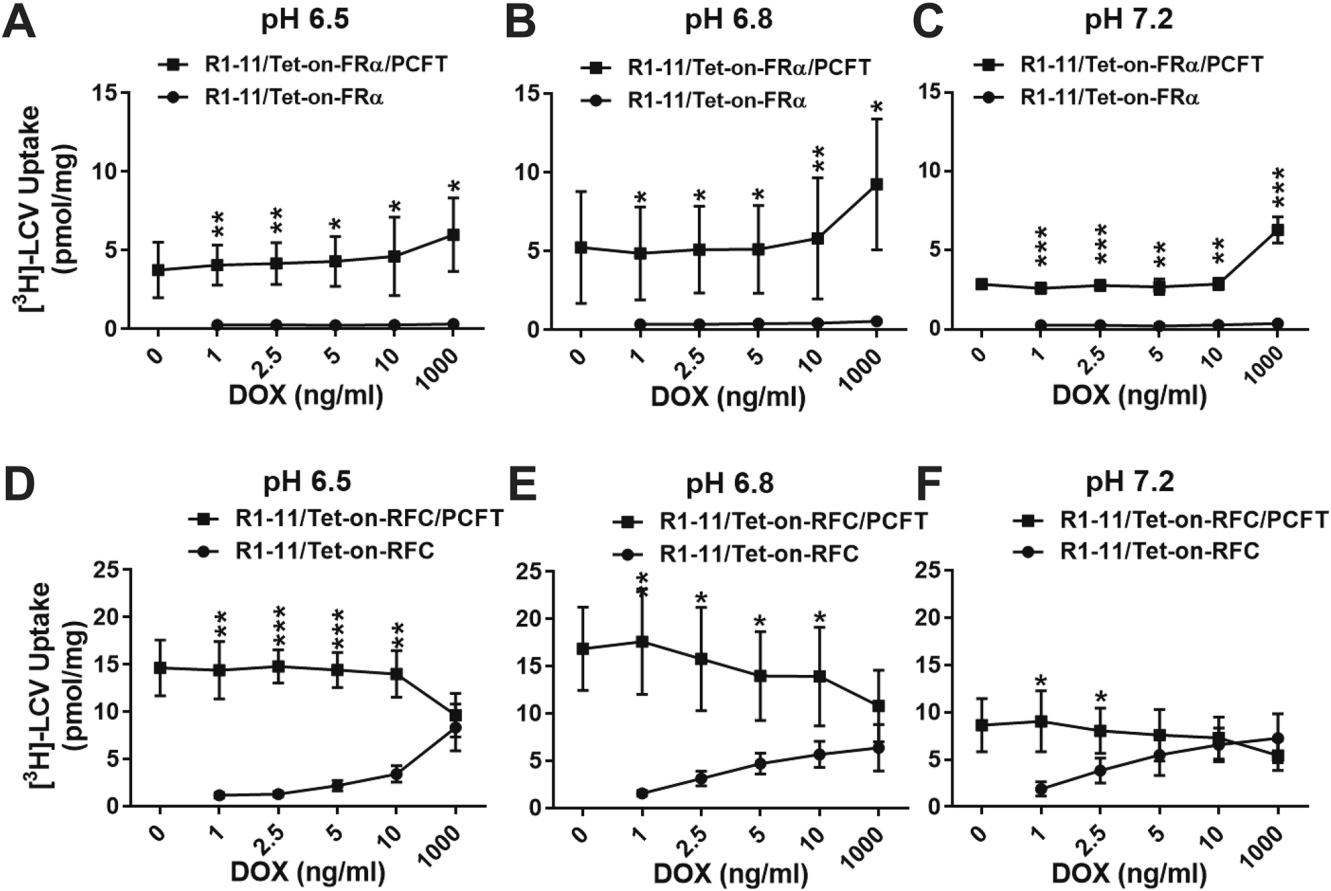
^*3*^*H-LCV accumulation by R1-11/Tet-on-FRα, R1-11/Tet-on-FRα/PCFT, R1-11/Tet-on-RFC and R1-11/Tet-on-RFC/PCFT cell line models.* R1-11/Tet-on-FRα, R1-11/Tet-on-FRα/PCFT, R1-11/Tet-on-RFC or R1-11/Tet-on-RFC/PCFT cells were plated in 60-mm dishes in complete FF RPMI 1640 medium containing 10% FBS for 24–48 h. The media was then replaced with complete FF RPMI 1640 medium (pH 6.5, 6.8 or 7.2) containing 10% dialyzed FBS, 25 nM [^3^H]LCV and a range of DOX (0, 1, 2.5, 5, 10, and 1000 ng/ml). Forty-eight hours later, the dishes were washed (2×) with acid buffer (10 mM sodium acetate, 150 mM NaCl, pH 3.5; for R1-11/Tet-on-FRα or R1-11/Tet-on-FRα/PCFT cells) and 3× with ice-cold PBS. The R1-11/Tet-on-RFC and R1-11/Tet-on-RFC/PCFT cells were washed 3× with ice-cold PBS only. The washed cells were solubilized in 0.5 N NaOH and radioactive contents and protein concentrations of the alkaline cell homogenates were determined. Intracellular radioactivity was calculated in units of pmol [^3^H]LCV per mg of cell protein. The [^3^H]LCV uptake results are presented as mean values plus/minus SDs from at least 3 experiments for incubations at pH 6.5 (**A**,**D**), pH 6.8 (**B**,**E**) and pH 7.2 (**C**,**F**). Statistical significance of [^3^H]LCV uptake values between the single and double transfectants at each DOX dosage was analyzed by an unpaired t test. An asterisk indicates a statistically significant difference between the mean values of [^3^H]LCV accumulation by single and double transfected cells at each DOX concentration (*p < 0.05; **p < 0.005; ***p < 0.0005).

Leucovorin (LCV) or (6R,S)5-formyl tetrahydrofolate, also known as folinic acid, is an analog of the synthetic folate form of folic acid. LCV is a mimic of the physiologic folate form (5-methyl tetrahydrofolate) which is more stable and thus suitable for in vitro experiments. We measured accumulation of [^3^H]LCV in R1-11/Tet-on-FRα (FRα only) and R1-11/Tet-on-FRα/PCFT (FRα and PCFT) cells induced with DOX (1, 2.5, 5, 10 and 1000 ng/ml) at pH 6.5, 6.8 and 7.2. Internalized [^3^H]LCV was distinguished from that bound to surface FRs by an acid (pH 3.5) wash prior to quantitation of cell-associated [^3^H]folates^12^. The contribution of FRα to [^3^H]LCV accumulation in R1-11/Tet-on-FRα cells was nominal regardless of pH (Fig. 5A–C). However, for the double-transfected R1-11/Tet-on-FRα/PCFT cells, uptake of [^3^H]LCV was markedly increased with similar levels at pH 6.5 and 6.8 and somewhat lower (~ 60%) levels at pH 7.2 (Fig. 5A–C). The increased [^3^H]LCV accumulation from 10 to 1000 ng/ml DOX parallels the increased PCFT transport flux measured over this range (Fig. 2F).

Analogous experiments were performed to compare [^3^H]LCV accumulations in R1-11/Tet-on-RFC (RFC only) and R1-11/Tet-on-RFC/PCFT (RFC and PCFT) cells. Overall, cellular folate accumulations were significantly higher in the double PCFT/RFC cells than in the single RFC transfected cells, especially at pH 6.5 and 6.8 (Fig. 5D,E), although RFC uptake of [^3^H]LCV was more pronounced at higher levels of RFC induction. With increasing pH, [^3^H]LCV accumulations in the R1-11/Tet-on-RFC/PCFT cells declined with increasing levels of RFC (Fig. 5D–F). This parallels changes seen in PCFT transport with increasing RFC (Fig. 3F). RFC uptake of [^3^H]LCV progressively increased from pH 6.5 to pH 7.2, paralleling the transport changes with pH (Fig. 4C), such that net uptake at pH 7.2 in R1-11/Tet-on-RFC cells with 1000 ng/ml DOX exceeded that of combined PCFT/RFC uptake in R1-11/Tet-on-RFC/PCFT cells (Fig. 5F). These results establish a predominant contribution of PCFT to cellular folate accumulation, particularly at acid pHs characterizing the tumor microenvironment.

### Transporter dynamics and anticancer therapy

We used cell proliferation assays with our engineered R1-11 cells to assess the impact on anti-tumor efficacies (as IC_50_ values) of classic and FR/PCFT-targeted antifolates of FRα alone (R1-11/Tet-on-FRα), and of FRα combined with constitutive PCFT expression (R1-11/Tet-on-FRα/PCFT). Cells were treated over 96 h with inhibitors with and without DOX (1000 ng/ml) induction. Inhibitors included: (1) classic antifolates with established patterns of transporter selectivity, including PMX (substrate for RFC and PCFT with PCFT > RFC and poor activity with FRα)^26,27^ and PT523 (RFC-specific without significant PCFT or FRα activity)^27,37^; and (2) targeted antifolates with dual specificities for FRα and PCFT but not RFC (AGF94)^28,32,33^, and for FRα without either PCFT or RFC (AGF102)^34^. As appropriate, excess FA (200 nM) was added with the inhibitors to confirm FRα-mediated inhibitory effects^12^.

Toward R1-11/Tet-on-FRα cells treated with DOX (FRα only), AGF94 and AGF102 were potent inhibitors, both with IC_50_ values < 3 nM; these increased ~ 128- and ~ 260-fold, respectively, in the presence of 200 nM FA (Table 1), establishing FR-specificity. For R1-11/Tet-on-FRα/PCFT cells without DOX (PCFT only), AGF94 was likewise active (IC_50_ = 1.3 nM) and was modestly impacted by excess FA (~ 10-fold); with FRα induction, potency increased slightly (~ 2-fold) and this was substantially reversed by FA (~ 350×). This establishes uptake of AGF94 by both PCFT and FRα, although with FRα induction, uptake by this mechanism predominated over PCFT.

**Table 1:** *Drug sensitivities (IC*_*50*_ *values) of R1-11/Tet-on-FRα and R1-11/Tet-on-FRα/PCFT (“double”) HeLa cells*.

Inhibitor	Selectivity	R1-11/Tet-on-FRα		R1-11/Tet-on-FRα/PCFT			
		1000 ng/ml DOX		0 ng/ml DOX		1000 ng/ml DOX	
		-FA	+ FA	-FA	+ FA	-FA	+ FA
		IC_50_ (nM)					
AGF94	FRα/PCFT	2.91 (0.82)	372 (100)	1.30 (0.65)	13.1 (8.0)	0.69 (0.13)	242 (125)
AGF102	FRα	2.84 (1.36)	738 (75)	476 (159)	836 (107)	46.3 (14.2)	727 (90)
PMX	RFC/PCFT	562 (108)	> 1000	11.9 (3.30)	42.6 (10.2)	20.4 (7.61)	61.8 (3.23)
PT523	RFC	177 (27)	330 (98)	> 1000	> 1000	212 (83.3)	> 1000

The cells were plated in 96-well culture plates (4,000 cells/well; 200 μl/well) with complete FF RPMI 1640 including 10% dialyzed FBS, 2 mM l-glutamine, and antibiotics, supplemented with 25 nM LCV. Drugs were added, with concentrations from 1 to 1000 nM for AGF102, AGF94, PMX, and PT523, in the presence of DOX (1000 ng/ml). Cells were incubated from 96 to120 hours (depending on the growth of the cell models) at 37 °C in a CO_2_ incubator. Cell viabilities were measured with a fluorescence-based viability assay (CellTiter-Blue; Promega) and a fluorescence plate reader (emission at 590 nm, excitation at 560 nm) for calculating the drug concentrations that inhibit growth by 50% (IC_50_). To demonstrate FRα-mediated drug uptake, excess (200 nM) FA was added to parallel cultures. Results are shown as mean IC_50_ values +/− standard errors (in parentheses) from 3 to 9 separate experiments.

R1-11/Tet-on-FRα/PCFT cells in the absence of FRα (no DOX) showed minimal sensitivity to AGF102 (IC_50_ ~ 476 nM) that increased ~ 10-fold (IC_50_ ~ 46.4 nM) with induction of FRα (Table 1). Interestingly, the IC_50_ value for AGF102 with combined expression of FRα and PCFT (R1-11/Tet-on-FRα/PCFT with DOX) was ~ 16-fold higher than that for FRα alone (R1-11/Tet-on-FRα with DOX) (Table 1). This establishes an antagonistic effect due to the presence of PCFT, likely attributable to elevated intracellular folates (Fig. 5A–C). While PMX sensitivity was modest in the absence of PCFT (562 nM) indicating its poor FR substrate activity, this substantially increased (~ 50-fold decreased IC_50_) in the presence of PCFT (R1-11/Tet-on-FRα/PCFT). In contrast, inhibition by PT523 was low and independent of the PCFT or FR expression (Table 1).

**Figure 6:**
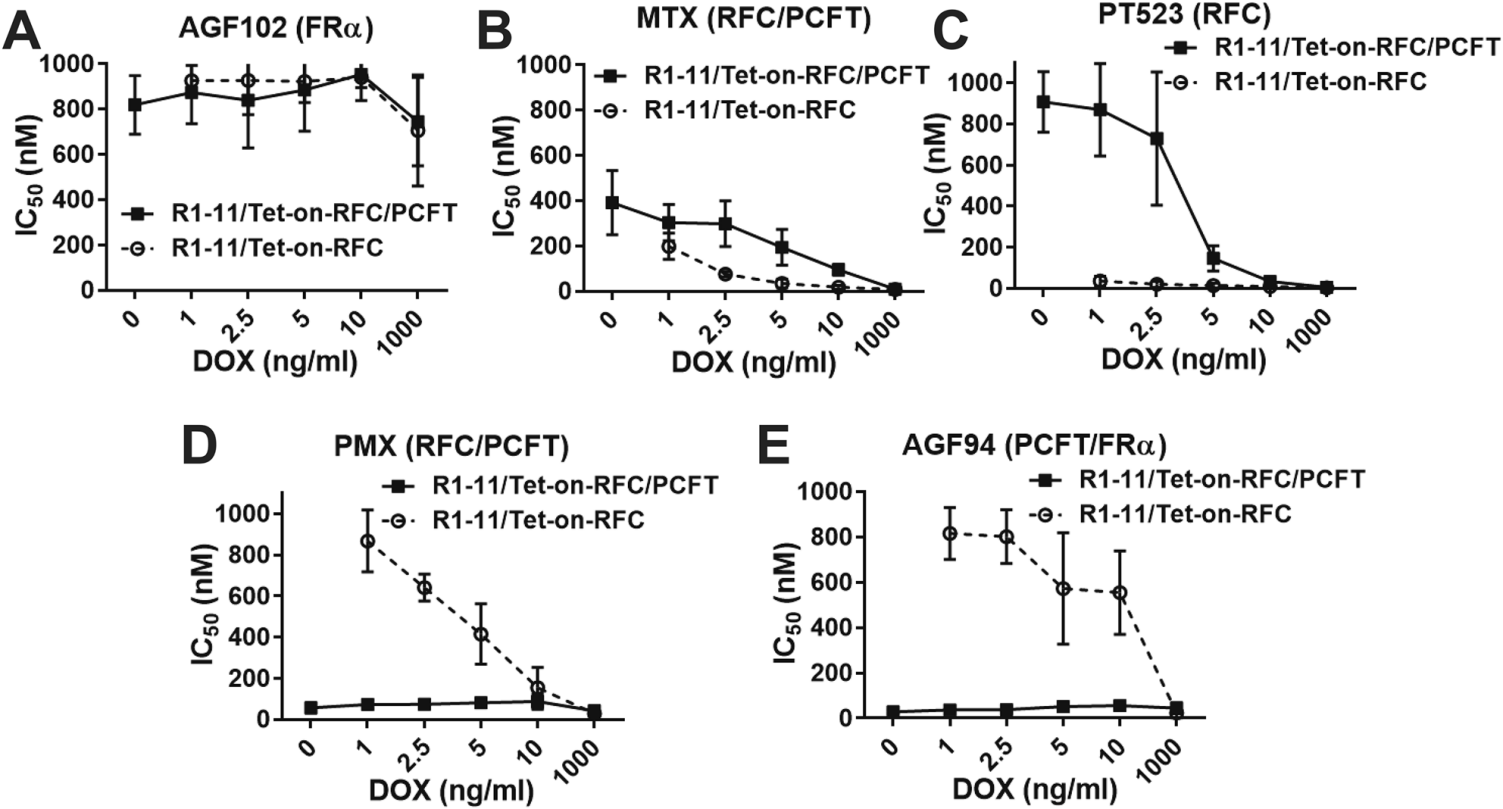
*Impact of RFC/PCFT redundancy on drug sensitivity.* R1-11/Tet-on-RFC or R1-11/Tet-on-RFC/PCFT cells were plated in 96-well culture plates (4000 cells/well; 200 μl/well) with complete FF RPMI 1640 including 10% dialyzed FBS supplemented with 25 nM LCV. Drugs with different transport specificities were added, with concentrations from 1 to 1000 nM for AGF102 (**A**), MTX (**B**), PT523 (**C**), PMX (**D**), and AGF94 (**E**). Cells were treated over a range of DOX concentrations (0, 1, 2.5, 5, 10 and 1000 ng/ml) and incubated from 96 to120 h (depending on the growth of the cell models) at 37 °C in a CO_2_ incubator. Cell viabilities were measured with a fluorescence-based viability assay (CellTiter-Blue; Promega) and a fluorescence plate reader (emission at 590 nm, excitation at 560 nm) for calculating the drug concentrations that inhibit growth by 50% (IC_50_). Results are shown as mean IC_50_ values + /− SDs from 4 to 15 separate experiments.

We measured sensitivities to classic and targeted antifolates by proliferation assays with the R1-11/Tet-on-RFC and R1-11/Tet-on-RFC/PCFT cells (Fig. 6). Cells were left untreated, or were treated with range of DOX concentrations to induce RFC and assayed for proliferation in the presence or absence of the inhibitors. As expected, the FR-selective inhibitor AGF102 was inactive toward both cell lines (IC_50_s > 600 nM) (Fig. 6A). MTX and PT523 inhibited proliferation of R1-11/Tet-on-RFC with decreasing IC_50_s accompanying increasing levels of RFC induction (Fig. 6B,C, respectively). For both compounds, constitutive PCFT expression (R1-11/Tet-on-RFC/PCFT) dramatically decreased inhibition, likely due to elevated intracellular folate pools (Fig. 5D–F). Differences in the inhibitions for MTX and PT523 between the R1-11/Tet-on-RFC and R1-11/Tet-on-RFC/PCFT cells reflected relative substrate activities for RFC and PCFT for MTX (RFC ≈ PCFT) versus PT523 (RFC >> PCFT).

For PMX and AGF94 (Fig. 6D,E, respectively), both of which prefer PCFT over RFC for transport, in vitro efficacy was modest (high IC_50_) over a range of RFC in the absence of PCFT (R1-11/Tet-on-RFC). There was a progressive decrease in IC_50_ values with increasing RFC for PMX, and significant inhibition was detected only at the highest level of RFC for AGF94. For both inhibitors, inhibition dramatically increased (decreased IC_50_) with constitutive expression of PCFT (R1-11/Tet-on-RFC/PCFT). These results reflect differences in transporter specificities for AGF94 versus PMX, with PCFT over RFC selectivity for AGF94 exceeding that for PMX^33^.

## Discussion

This study explores the transport dynamics between the major folate transporters RFC, PCFT and FRα in relation to folate homeostasis and anti-tumor efficacies of classic and tumor-targeted antifolates. Our results provide several novel insights.

We established a “cross-talk” between the folate transporters that manifests as decreased PCFT transport activity but not PCFT protein accompanying increased RFC levels, and enhanced PCFT transport with increasing levels of FRα. Although the mechanistic basis for these findings for RFC and PCFT is unclear, PCFT has been shown to facilitate FR-mediated uptake by mediating endosomal efflux^42^, although this is not obligatory^29,43^. We showed that cellular accumulations of extracellular folates from LCV are determined by the type and relative levels of the major folate transport systems, with PCFT and RFC prevailing over FRα, depending on the extracellular pH. Importantly, these results establish PCFT as a remarkably robust means of folate uptake under pH conditions (pH 6.5–6.8) commonly associated with the tumor microenvironment^39,40^. Interestingly, even at the most acidic pH conditions tested (i.e., pH 5.5), uptake by RFC was detectable.

Based on patterns of growth inhibition, our results establish transport redundancy for RFC and PCFT in the uptake of PMX, whereby PCFT compensates for low levels of RFC. For PMX, uptake by PCFT predominated and was independent of the level of RFC expressed. However, in the absence of PCFT, PMX growth inhibition increased linearly with RFC. While uptake of MTX by RFC (resulting in inhibition of cell proliferation) prevailed over PCFT accompanying a wide range of RFC expression, growth inhibition was clearly antagonized by PCFT. This effect of PCFT is likely due to elevated intracellular folates derived from LCV, as noted above, that compete for binding to the target enzyme and for polyglutamate synthesis. Not surprisingly, this was greatest at the lowest levels of RFC. Analogous results were seen with PT523. While the impact of RFC transport of extracellular folates on efficacy of PCFT-transported PMX has been described^44^, an analogous effect of PCFT on RFC substrates has not been reported. From patterns of AGF94 growth inhibition, redundancy was also seen for PCFT and FRα, although when both PCFT and FRα were expressed, uptake by FRα appeared to predominate based on patterns of competition by FA, although this may also in part reflect the impact of elevated intracellular folates in response to elevated FRα on target inhibition and AGF94 polyglutamylation, as described above. Our results with AGF102^34^ demonstrate tumor targeting of this novel cytotoxic thienopyrimidine analog was nearly exclusively by FRα. However, similar to MTX and PT523 which are transported predominately by RFC, anti-tumor efficacy of AGF102 was adversely effected by PCFT, likely due to its ability to transport extracellular folates even at pHs (6.5 and 6.8) far from its pH optimum.

Cellular levels of folates and folate analogs accumulated are the net result of both influx (FRα, RFC and PCFT) and efflux (ABCC1-5, BCRP/ABCG2) systems^45,46^, and this is a dynamic process. We found for ABCC1-5, as well as BCRP, gene expression differences with and without DOX were mostly modest, with differences within a 20–30% range and somewhat greater differences for ABCC2 in the Tet-on-FRα cells, and for ABCC2 and ABCC3 in the Tet-on-FRα/PCFT cells (Fig. S1, Supplement). Although the transport specificities by the major folate efflux systems for the novel antifolates reported here have not been studied, their slightly increased levels in the absence of DOX induction could be envisaged to contribute to modestly decreased levels of intracellular folates and antifolates compared to levels in the presence of DOX. Of course, the impact of efflux vis á vis influx in net (anti)folate accumulation would depend on relative transporter expression levels and rates of uptake and efflux, along with the extent of polyglutamate synthesis for the (anti)folate forms, as polyglutamyl (anti)folates are not substrates for efflux^25,47^.

Relative expression of the major folate uptake systems may provide prognostic markers for clinical use of cytotoxic folate analogs. For the classical antifolates MTX and PMX which are both RFC and PCFT substrates (albeit with different specificities), high expression of PCFT over RFC in tumors may negatively impact anti-tumor efficacy of MTX, although this could benefit PMX efficacy. For tumor-targeted antifolates such as AGF94, which target both FRα and PCFT, FRα or PCFT expression would be sufficient for drug uptake and anti-tumor efficacy (thus compensating for the loss of one system), although expression of both FRα and PCFT would certainly enhance anti-tumor activity. Conversely, for exclusively FRα-selective agents, i.e., AGF102, anti-tumor efficacy would be adversely impacted by PCFT expression.

Collectively, our findings describe a previously unrecognized interplay among the major folate transport systems that depends on transporter levels and extracellular pH, and that determines their contributions to the uptake and anti-tumor efficacies of targeted and untargeted antifolates.

## Materials and methods

### Reagents

Syntheses of AGF94^28^ and AGF102^34^ were previously described. PMX (Alimta) was obtained from Eli Lilly and Company (Indianapolis, IN). PT523^37^ was a gift of Dr. Andre Rosowsky (Harvard University; Boston, MA). MTX and LCV were obtained from the Drug Development Branch, National Cancer Institute (Bethesda, MD). [^3^H]MTX (20 Ci/mmol), [^3^H]FA (27.2 Ci/mmol), and [^3^H]LCV (15.6 Ci/mmol) were purchased from Moravek Biochemicals (Brea, CA). Other chemicals were obtained from commercial sources in the highest available purities.

### Construction of human RFC, PCFT and FRα expression constructs

To prepare a Tet inducible expression construct for RFC with a C-terminal HA-tag^48,49^ (at position 591) (Tet-on-RFC^HA^), a DNA fragment encoding RFC (HA sequence at the 3′ terminus) was generated from RFC^HA^/pTre2hyg (previously generated in our laboratory to induce expression of HA-tagged RFC in the pTre2hyg vector) by MluI and BglII digestions, and subcloned into the MluI and BglII sites in the pTetOne Vector (Clontech Laboratories, Mountainview, CA) (Tet-on-RFC^HA^).

A Tet-inducible expression construct of FRα with an N-terminal HA-tag (Tet-on-^HA^FRα) was prepared by three steps. (1) A DNA fragment encoding FRα was PCR-amplified from FRα/pcDNA3^43^ with addition of a MluI site at the 5′ end and a BglII site at the 3′ end. (2) Following digestions with MluI and BglII, the fragment was subcloned into the pTetOne Vector between the MluI and BglII sites (Tet-on-FRα). (3) An HA tag was inserted between amino acids 29 and 30 of FRα by mutagenesis PCR, generating Tet-on-^HA^FRα.

To express carboxyl terminal HA-tagged PCFT (PCFT^HA^) at moderate levels under control of a TK promoter^50,51^, we first engineered the mammalian expression vector pcDNA3.1TK/Zeo(+) by replacing the CMV promoter in pcDNA3.1/Zeo(+) (Invitrogen, Carlsbad, CA) (by digestion with NruI and AgeI) with a HSV-TK promoter from pGL4.74[hRluc/TK] (Promega, Madison, WI) (digested with KpnI (blunted by Klenow polymerase) and AgeI). A PCFT^HA^ DNA fragment was generated by digesting PCFT^HA^/pcDNA3.1/Zeo(+) (previously generated in our laboratory to express HA-tagged PCFT^52^ in pcDNA3.1/Zeo(+) vector) with HindIII and XbaI, and subcloning into pcDNA3.1TK/Zeo(+) at the HindIII and XbaI sites (generates PCFT^HA^/pcDNA3.1TK/Zeo(+)).

All constructs were confirmed by Sanger sequencing (Genewiz; South Plainfield, NJ). Primers used for generating the mutations, deletions and insertions are summarized in Table S1 and were purchased from Invitrogen (Carlsbad, CA).

### Cell culture

RFC-, FR-, and PCFT-null HeLa R1-11 cells^53^ were transfected with Lipofectamine 2000 (Invitrogen, Carlsbad, CA), as described previously^33^. The Tet-on-^HA^FRα or Tet-on-RFC^HA^ constructs (above), together with a linear puromycin marker (Clontech, Mountain View, CA), were transfected into R1-11 cells, followed by selection with puromycin (2000 ng/ml) until a stable cell mixture resulted. The stable cell clones were plated in a 96-well plate without or with DOX (1000 ng/ml) to induce protein expression (2 wells per condition), for screening of FRα or RFC-expressing stable clones by “in-cell” Westerns per the manufacturer’s protocol (LI-COR Biosciences, Lincoln, NE) with anti-HA monoclonal antibody (Covance, Emeryville, CA). A number of positive clones were isolated for each stable cell mixture from which we selected clone #15 for R1-11/Tet-on-FRα and clone #36 for R1-11/Tet-on-RFC for subsequent analyses. The PCFT^HA^/pcDNA3.1TK/Zeo(+) construct (above) was transfected into R1-11/Tet-on-FRα #15 or R1-11/Tet-on-RFC #36 cells; single clones were selected and isolated as described above. We selected R1-11/Tet-on-FRα/PCFT #11 and R1-11/Tet-on-RFC/PCFT #1 clones for our study (hereafter, simply R1-11/Tet-on-FRα/PCFT and R1-11/Tet-on-RFC/PCFT, respectively). The “single stable” Tet-On cell lines R1-11/Tet-on-FRα and R1-11/Tet-on-RFC were selected with puromycin (2000 ng/ml) and “double stable” R1-11/Tet-on-FRα/PCFT and R1-11/Tet-on-RFC/PCFT cell lines were selected with both puromycin (2000 ng/ml) and zeocin (1000 µg/ml).

Parental R1-11 cells and the engineered Tet-On cell lines in a R1-11 background were routinely cultured in complete RPMI 1640 medium, containing 10% fetal bovine serum (FBS; Sigma, St. Louis, MO), 2 mM l-glutamine, 100 units/ml penicillin, and 100 µg/ml streptomycin, in a humidified atmosphere at 37 °C in the presence of 5% CO_2_ and 95% air with antibiotics (above).

Prior to functional characterization (e.g., transport, [^3^H]FA binding, etc.) (below) and cell proliferation assays with classic and targeted antifolates, the engineered cell lines were cultured in folate-free (FF) RPMI 1640 medium containing 10% FBS, 2 mM l-glutamine, 100 units/ml penicillin, and 100 µg/ml streptomycin for 7–10 days.

For cell proliferation assays, cells were plated in 96-well culture plates (4000 cells/well; 200 μl/well) with complete FF RPMI 1640 with 10% dialyzed FBS, supplemented with 25 nM LCV. Cell proliferation assays for R1-11/Tet-on-FRα or R1-11/Tet-on-FRα/PCFT cells were performed with and without DOX (1000 ng/ml), whereas those for R1-11/Tet-on-RFC or R1-11/Tet-on-RFC/PCFT cells were performed without DOX or with a range of DOX concentrations (1, 2.5, 5, 10, and 1000 ng/ml). Inhibitors (AGF102, AGF94, MTX, PMX, and PT523) were added at concentrations from 1 to 1000 nM. Cells were incubated for 96–120 h (depending on the cell line) at 37 °C in a CO_2_ incubator. Cell viabilities were measured with a fluorescence-based assay (CellTiter-Blue; Promega, Madison, WI) and a fluorescence plate reader (emission 590 nm, excitation 560 nm) for calculating the inhibitor concentrations that decreased growth by 50% (IC_50_)^43^. To confirm FRα-mediated drug uptake, excess (200 nM) FA was added to parallel cultures. Under these conditions, cellular uptake by PCFT was unaffected^54^.

### [^3^H]LCV accumulation studies

For [^3^H]LCV accumulation studies, the engineered Tet-On stable cell lines (R1-11/Tet-on-RFC, R1-11/Tet-on-FRα, R1-11/Tet-on-FRα/PCFT, R1-11/Tet-on-RFC/PCFT) were plated in 60-mm dishes in complete FF RPMI 1640 with 10% FBS for 24–48 h at 37 °C in 5% CO_2_/95% air. The medium was replaced with complete FF RPMI 1640 with 10% dialyzed FBS (pH 6.5, 6.8 or 7.2) including 25 nM [^3^H]LCV and a range of DOX concentrations (0, 1, 2.5, 5, 10, and 1000 ng/ml). After 48 h, for R1-11/Tet-on-RFC and R1-11/Tet-on-RFC/PCFT cells, the dishes were washed 3× with ice-cold Dulbecco’s phosphate-buffered saline (PBS), then solubilized in 0.5 N NaOH. The R1-11/Tet-on-FRα and R1-11/Tet-on-FRα/PCFT cells were washed with ice-cold PBS (2×), acetate buffer (10 mM sodium acetate, 150 mM NaCl, pH 3.5) (2×), and PBS (3×), then solubilized in 0.5 N NaOH. Aliquots of the alkaline extracts were assayed for radioactivity and proteins^55^. Folates derived from [^3^H]LCV were expressed in units of pmol per mg of cell protein.

### Transport assays

Transport assays in monolayer cultures were performed for PCFT^41,52^ and RFC^56,57^. Cells (R1-11/Tet-on-RFC, R1-11/Tet-on-FRα/PCFT, R1-11/Tet-on-RFC/PCFT) were plated in 60-mm dishes in complete FF RPMI 1640 medium with 10% FBS. After 24 h, DOX (0, 1, 2.5, 5, 7.5, 10, 25, 50, and 1000 ng/ml) was added. After an additional 48 h, cellular uptake of [^3^H]MTX (at 0.5 μM) was measured over 2 min at 37 °C in MES-buffered saline (MBS) (20 mM MES, 140 mM NaCl, 5 mM KCl, 2 mM MgCl_2_, and 5 mM glucose, pH 5.5) (for PCFT) or in anion-free buffer (20 mM Hepes and 235 mM sucrose, pH 7.3) (for RFC). The dishes were washed 3 × with ice-cold PBS; the cells were solubilized with 0.5 N NaOH and assayed for radioactivity and proteins^55^. Intracellular radioactivity was calculated in units of pmol [^3^H]MTX per mg of cell protein.

To concurrently profile uptake via RFC and PCFT (with R1-11/Tet-on-RFC and R1-11/Tet-on-RFC/PCFT cells) from pH 5.5–7.4, cells were plated in 60-mm dishes in complete FF RPMI 1640 medium with 10% FBS. After 24 h, DOX (10 ng/ml final) was added. Following an additional 48 h, uptake was measured over 2 min at 37 °C with 0.5 µM [^3^H]MTX in MBS (for pH 5.0, 6.0 and 6.5) and Hepes-buffered saline (HBS) (20 mM Hepes, 140 mM NaCl, 5 mM KCl, 2 mM MgCl_2_, and 5 mM glucose; for pH 6.8, 7.0, 7.2 and 7.4) buffers. To distinguish uptake by RFC vis á via PCFT, cells were treated in parallel with PT523^37^ and AGF94^28^, respectively (both at 10 µM), to inhibit the individual transporters. Cells were washed at 4 °C with PBS (3×), then solubilized in 0.5 N NaOH for determinations of radioactivity and proteins^55^. Intracellular radioactivity was calculated in units of pmol [^3^H]MTX per mg of cell protein.

### [^3^H]FA binding assays

[^3^H]FA binding to surface FRα as a measure of FRα levels was determined as described previously^12^. Briefly, R1-11/Tet-on-FRα, and R1-11/Tet-on-FRα/PCFT cells were plated in 60-mm dishes in complete FF RPMI 1640 with 10% FBS. After 24 h, DOX (0, 1, 2.5, 5, 7.5, 10, 25, 50, and 1000 ng/ml) was added. The dishes were incubated for 48 h, then rinsed at 4° C with PBS (3×), acetate buffer (above) (2×), and HBS (3×). The cells were incubated in HBS with [^3^H]FA (50 nM) for 15 min at 0 °C, then rinsed with ice-cold HBS (3×) and solubilized with 0.5 N NaOH. The alkaline homogenates were measured for radioactivity and proteins^55^. FRα-bound [^3^H]FA was calculated as pmol [^3^H]FA/mg protein.

### Western blot analysis

Cells (R1-11/Tet-on-RFC, R1-11/Tet-on-RFC/PCFT, R1-11/Tet-on-FRα and R1-11/Tet-on-FRα/PCFT) were plated in 60-mm dishes in complete FF RPMI 1640 containing 10% FBS. After 24 h, DOX (0, 1, 2.5, 5, 7.5, 10, 25, 50, and 1000 ng/ml) was added. After 48 h, cells were washed with PBS and disrupted in 10 mM Tris–HCl (pH 7) in the presence of cOmplete Protease Inhibitor Cocktail (Roche Diagnostics, Indianapolis, IN) by sonication. Cell debris was removed by centrifugation (1,800 rpm, 5 min); the particulate membrane fraction was prepared by high-speed centrifugation (37,000×*g*, 30 min). The membrane pellet was solubilized (1% SDS in 10 mM Tris–HCl (pH 7) with protease inhibitors). Membrane proteins (50 μg) were electrophoresed on 7.5% Tris/glycine gels with SDS^58^ and transferred to polyvinylidene difluoride membranes (Thermo Scientific, Rockford, IL)^59^. As warranted (R1-11/Tet-on-RFC/PCFT cells), membrane proteins (50 μg) were deglycosylated with N-glycosidase F (1000 units; New England Biolabs, Ipswich, MA)^52^ before Western blotting. To detect HA-tagged RFC, PCFT or FRα, anti-HA monoclonal antibody (Cat# MMS-101P-500; Covance, Emeryville, CA) was used. Mouse Na^+^/K^+^ ATPase (Cat# NB300-146; Novus Biologicals, Littleton, CO) was used as a loading control. IRDye800CW-conjugated goat anti-mouse IgG (Cat# 926-32210; LI-COR Biosciences, Lincoln, NE) was the secondary antibody. Membranes were scanned with an Odyssey infrared imaging system (LI-COR Biosciences, Omaha, NE).

### Statistical analysis

Statistical analyses were performed using GraphPad Prism version 6.07 for Windows (GraphPad Software, LaJolla, CA). Differences between two groups were statistically assessed using an unpaired or paired t test.

## Supplementary Information

Supplementary Information

## Abbreviations

AICARFTase: 5-Aminoimidazole-4-carboxamide ribonucleotide formyltransferase
BCRP/ABCG2: Breast cancer resistance protein
C1: One-carbon
DOX: Doxycycline
FA: Folic acid
FBS: Fetal bovine serum
FF: Folate-free
FR: Folate receptor
FRs: Folate receptors
GARFTase: Glycinamide ribonucleotide formyltransferase
GI: Gastrointestinal
HA: Hemagglutinin
HBS: Hepes-buffered saline
IC_50_: 50% Inhibitory concentration
LCV: Leucovorin
MBS: MES-buffered saline
MRP/ABCC: Multidrug resistance proteins
MTX: Methotrexate
PBS: Dulbecco’s phosphate-buffered saline
PCFT: Proton-coupled folate transporter
PMX: Pemetrexed
RFC: Reduced folate carrier
SD: Standard deviation
Tet: Tetracycline
TK: Thymidine kinase
