# Supplementary material for: Folate transporter dynamics and therapy with classic and tumor-targeted antifolates
Source: Sci Rep. 2021 Mar 18;11:6389. doi: 10.1038/s41598-021-85818-x (PMC7973545; doi:10.1038/s41598-021-85818-x)

# Folate Transporter Dynamics and Therapy with Classic and Tumor-targeted Antifolates

Carrie O'Connor[1], Adrianne Wallace-Povirk[1], Changwen Ning[1], Josephine Frühauf[1], Nian Tong[4], Aleem Gangjee[4], Larry H. Matherly[1,2,3,*], and Zhanjun Hou[1,3,*]

From the [1]Departments of Oncology and [2]Pharmacology (L.H.M), Wayne State University School of Medicine, Detroit, Michigan and the [3]Molecular Therapeutics Program, Barbara Ann Karmanos Cancer Institute; and the [4]Division of Medicinal Chemistry, Duquesne University, Pittsburgh, Pennsylvania

*To whom correspondence should be addressed: Zhanjun Hou, Ph.D, 421 E. Canfield, Detroit, MI 48201; Tel.: 313-578-4372; Fax: 313-578-4287; E-mail: houz@karmanos.org

Larry H. Matherly, Ph.D, 421 E. Canfield, Detroit, MI 48201; Tel.: 313-578-4280; Fax: 313-578-4287; E-mail: matherly@karmanos.org

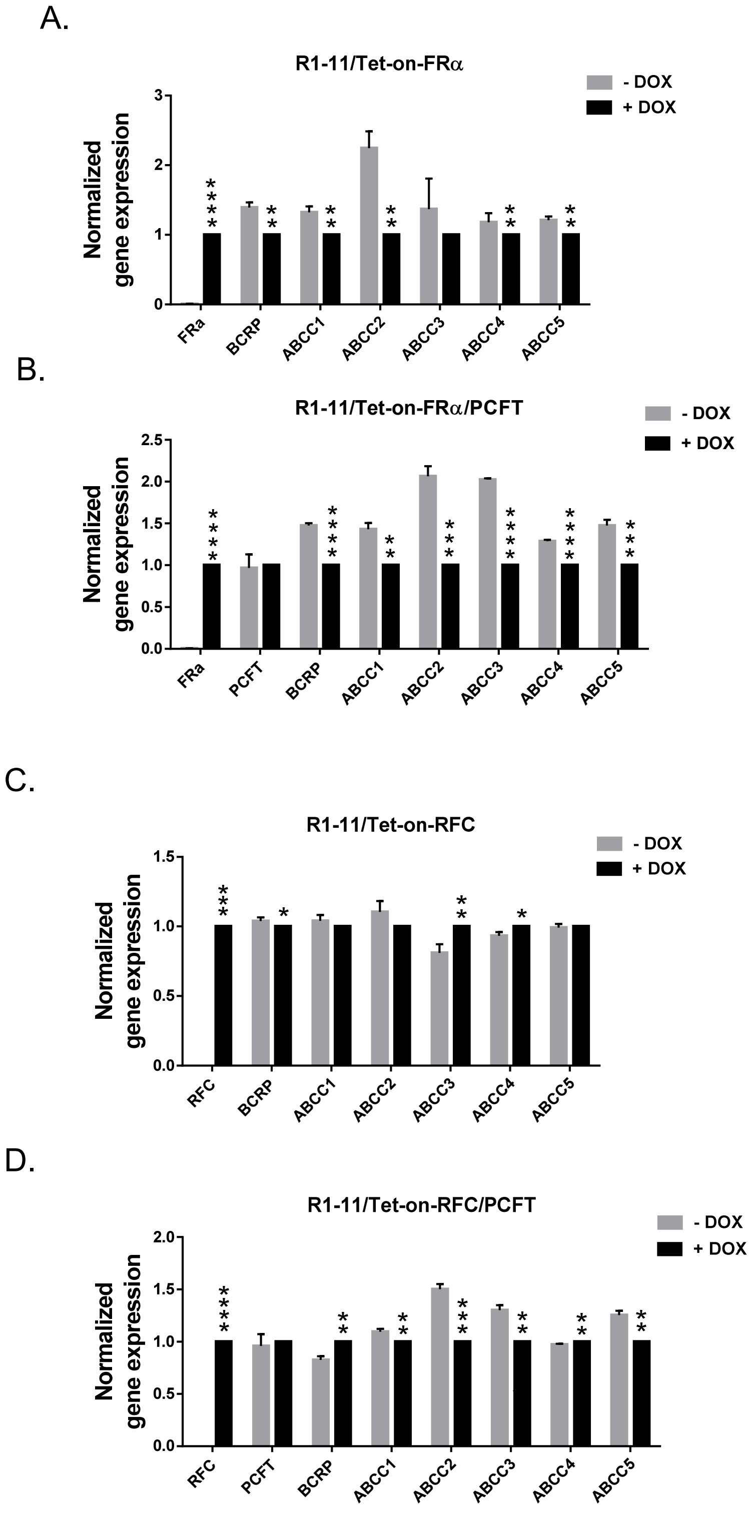


**Figure S1. Gene expression of ABCC1-5 and BCRP in single (Tet-on-FRα and Tet-on-RFC) and dual (Tet-on-FRα/PCFT, Tet-on-RFC/PCFT) models.** Transcript levels for ABCC1-5, BCRP and relevant folate uptake systems (FRα, PCFT or RFC) were measured in the Tet-on-FRα (A), Tet-on-FRα/PCFT (B), Tet-on-RFC (C) and Tet-on-RFC/PCFT (D) cell line models, with and without DOX (1000 ng/ml). Transcript levels of these genes were monitored by real-time RT-PCR

with a LightCycler 480 SYBR Green I Master kit. The transcript levels were normalized to that for GAPDH and/or β-actin. Results are presented as mean values ± standard errors from three to four different experiments. For statistics: ****$p$ < 0.0001; ***$p$ < 0.005; **$p$ < 0.01; *$p$ < 0.05.

**Table S1**. **Primers used for preparing various constructs.**

| **Primer use** | **Primer name** | **Primer sequence** |
|---|---|---|
| For cloning $^{HA}$FRα into pTetOne | hFRa-pTet/s(MluI) | aactcacgcgtagacatggctcagcggatg |
| For cloning $^{HA}$FRα into pTetOne | hFRa-pT/as(BglII) | aactcagatcttcaagcgtaatccggaacatcg |
| For cloning $^{HA}$FRα into pTetOne | HAhFRa/pTet(*)_S | gctgtggctgctcagctgatacccatacgatgttc |
| For cloning $^{HA}$FRα into pTetOne | HAhFRa/pTet(*)_AS | gaacatcgtatgggtatcagctgagcagccacagc |
| For cloning $^{HA}$FRα into pTetOne | HAhFRa/pTet(HA)_S | aaggattgcatgggcctacccatacgatgttccggattacgctaggactgag cttctca |
| For cloning $^{HA}$FRα into pTetOne | HAhFRa/pTet(HA)_AS | tgagaagctcagtcctagcgtaatccggaacatcgtatgggtaggcccatg caatcctt |
| For cloning of pcDNA3.1TKZeo(+) | 3.1ZeoCMV3AgeI_S | agagaacccactgcttactggcttataccggttaatacgactcactatággga gacc |
| For cloning of pcDNA3.1TKZeo(+) | 3.1ZeoCMV3AgeI_AS | ggtctccctatagtgagtcgtattaaccggtataagccagtaagcagtgggtt ctct |
| For cloning of pcDNA3.1TKZeo(+) | pGL4.74TK3AgeI_S | gaccctgcagcgacccgcttaaaccggtggcaatccggt |
| For cloning of pcDNA3.1TKZeo(+) | pGL4.74TK3AgeI_AS | accggattgccaccggtttaagcgggtcgctgcagggtc |

# Figure S2. Full blot of Figure 2A Upper Panel

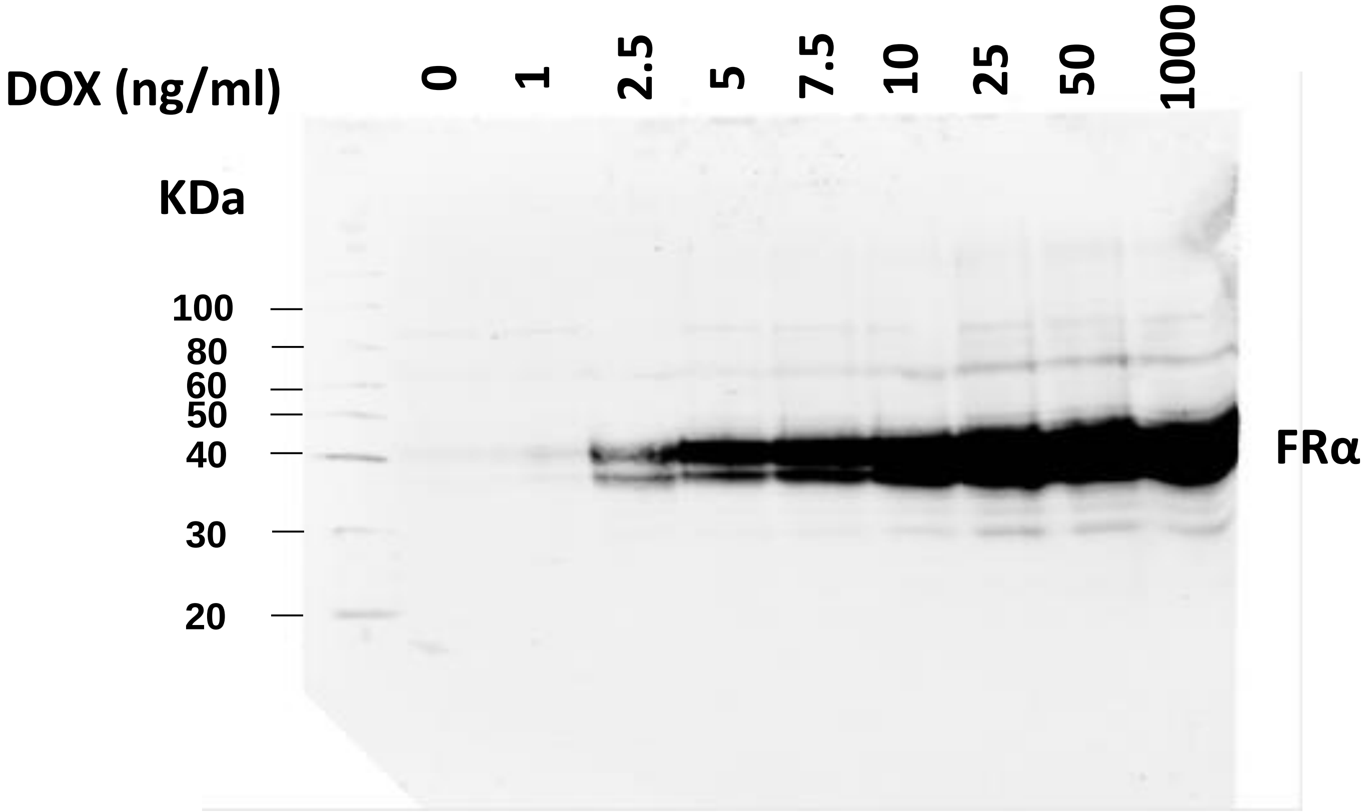

# Figure S3. Full blot of Figure 2A Lower Panel

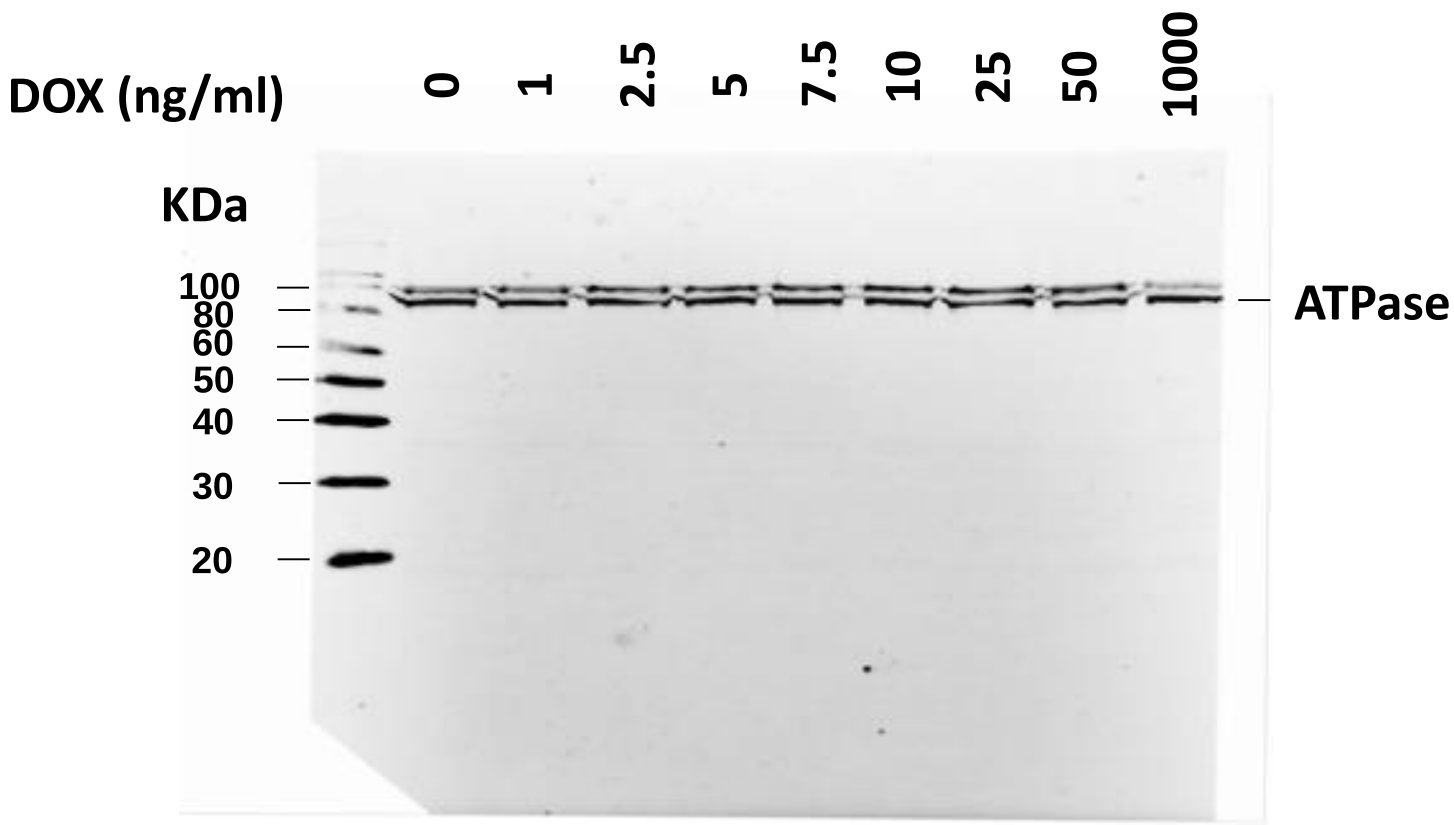

# Figure S4. Full blot of Figure 2B Upper Panel

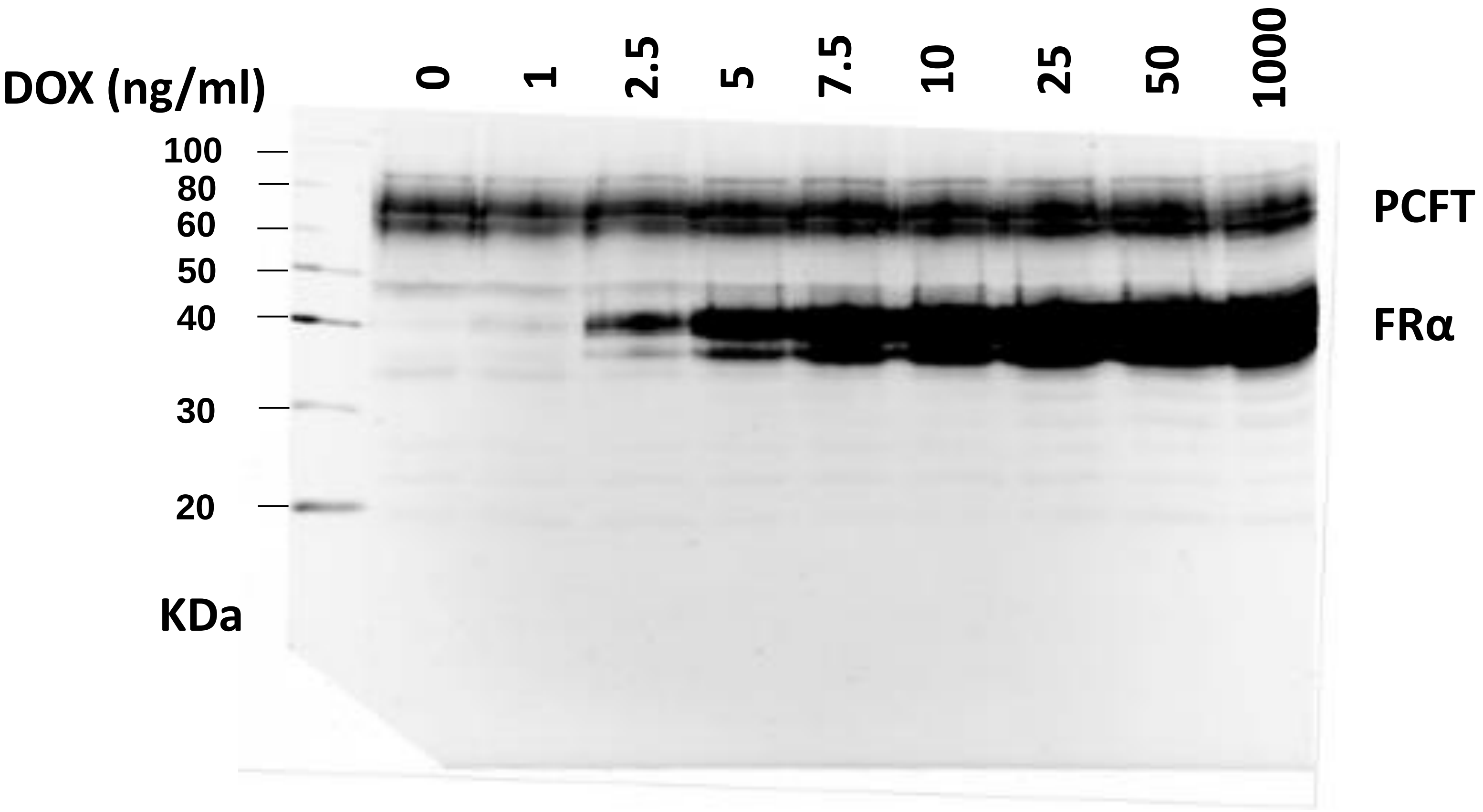

# Figure S5. Full blot of Figure 2B Lower Panel

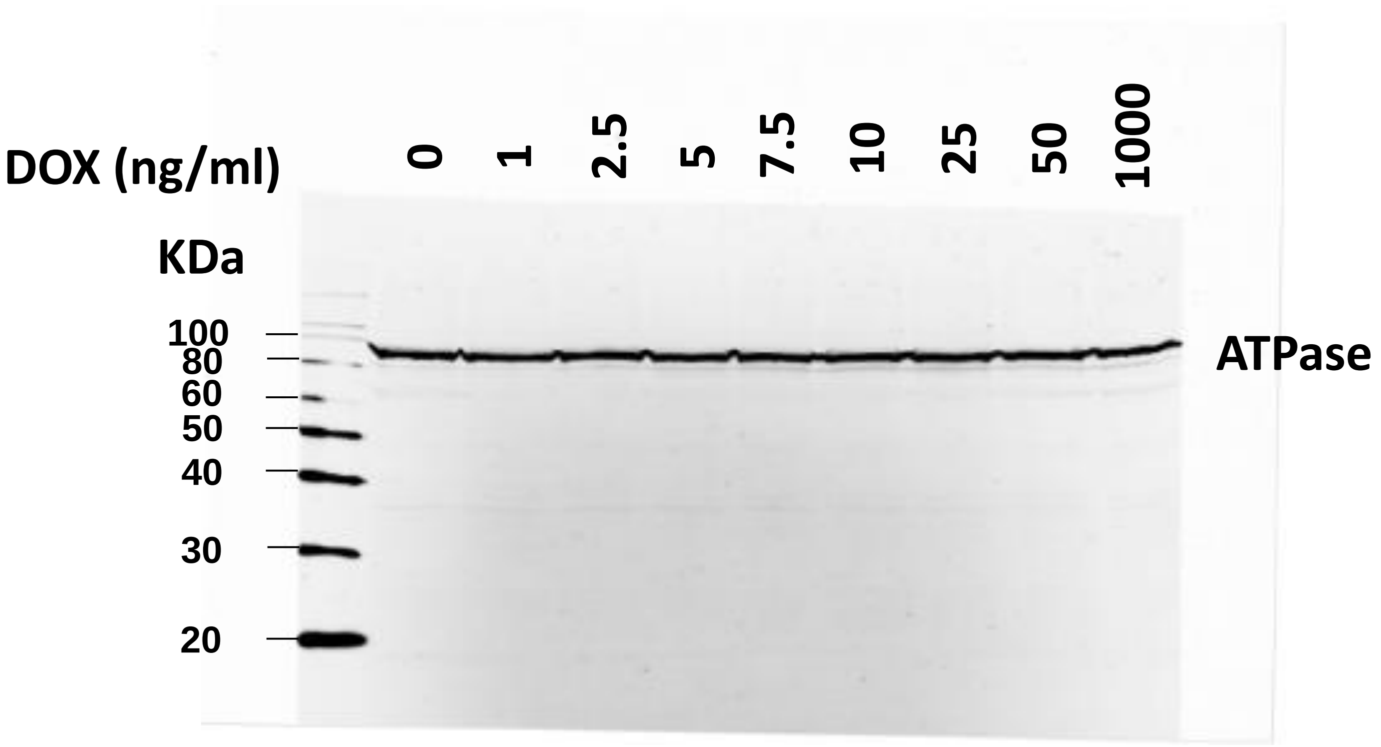

# Figure S6. Full blot of Figure 3A Upper Panel

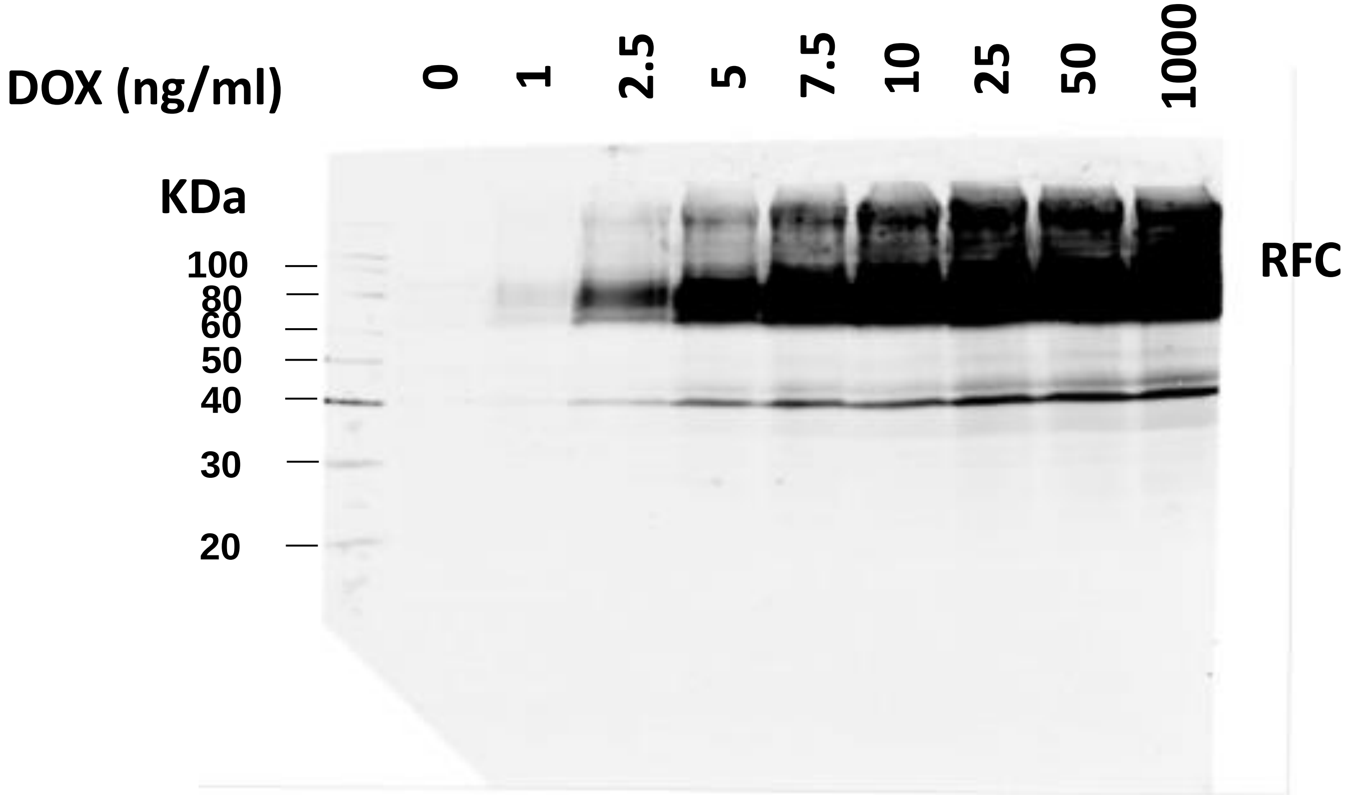

# Figure S7. Full blot of Figure 3A Lower Panel

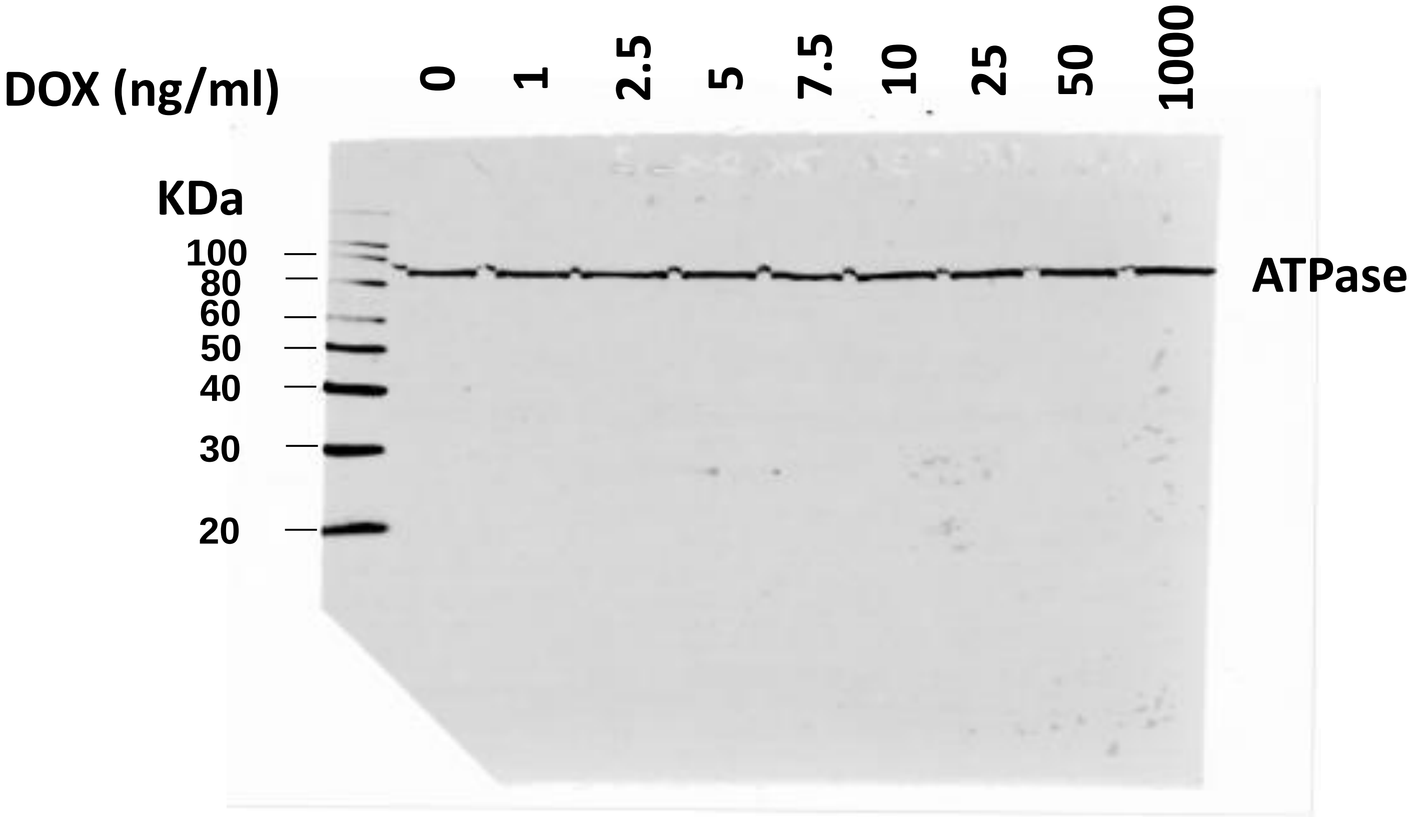

# Figure S8. Full blot of Figure 3B Upper Panel

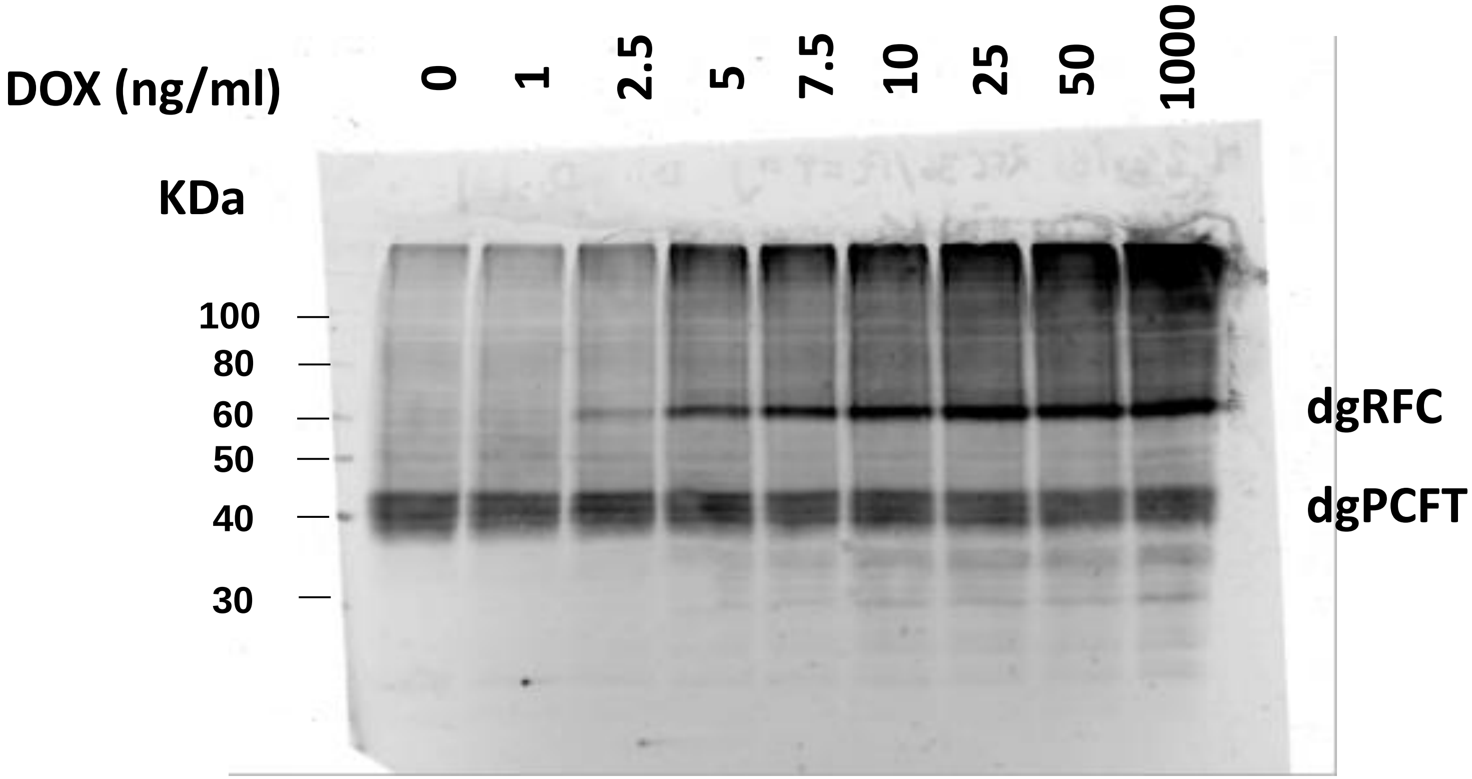

# Figure S9. Full blot of Figure 3B Lower Panel

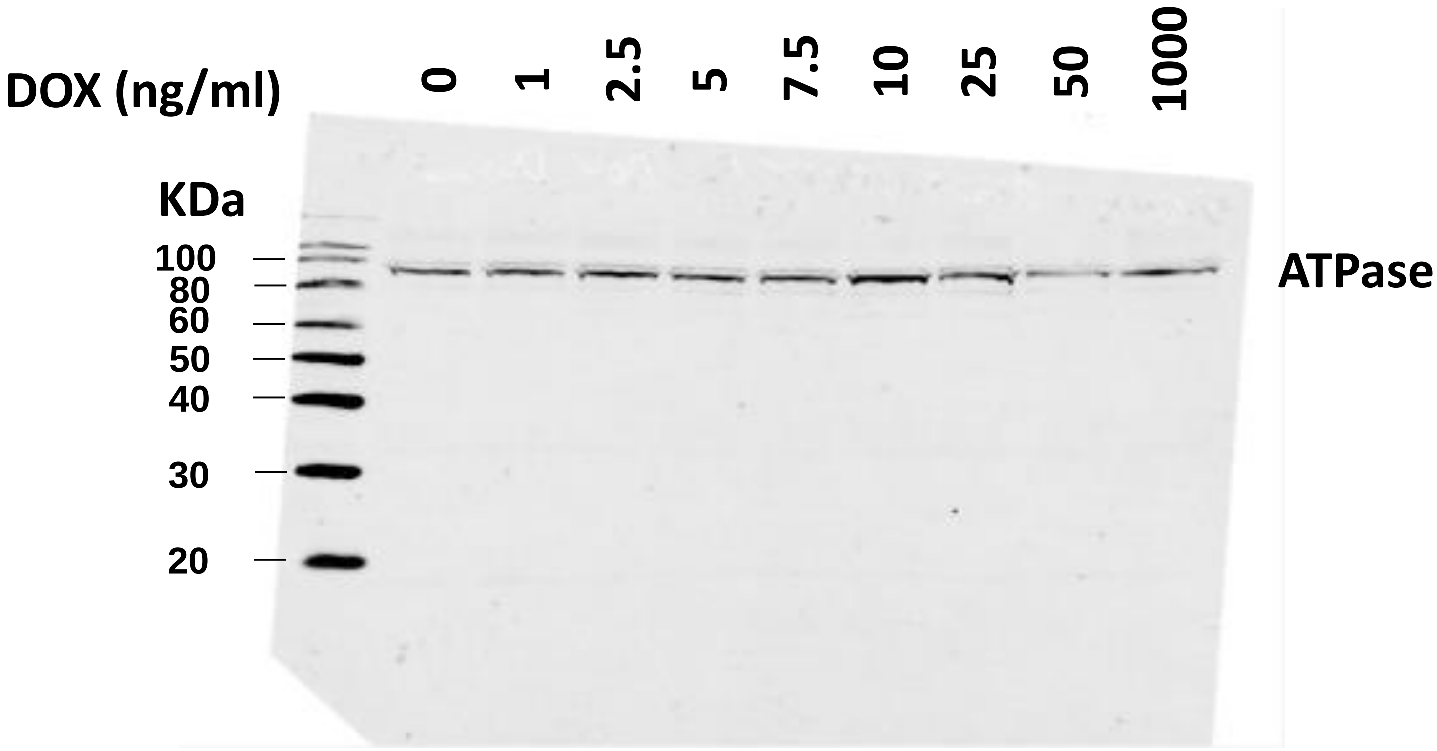

Supplement: Supplementary file 1 — Supplementary Information [file 41598_2021_85818_MOESM1_ESM.pdf]
